# Membrane protein provision controls prothylakoid biogenesis in tobacco etioplasts

**DOI:** 10.1093/plcell/koae259

**Published:** 2024-09-25

**Authors:** Bingqi Li, Tegan Armarego-Marriott, Łucja Kowalewska, Wolfram Thiele, Alexander Erban, Stephanie Ruf, Joachim Kopka, Mark Aurel Schöttler, Ralph Bock

**Affiliations:** Max Planck Institute of Molecular Plant Physiology, 14476 Potsdam-Golm, Germany; Max Planck Institute of Molecular Plant Physiology, 14476 Potsdam-Golm, Germany; Faculty of Biology, Department of Plant Anatomy and Cytology, University of Warsaw, 02-096 Warsaw, Poland; Max Planck Institute of Molecular Plant Physiology, 14476 Potsdam-Golm, Germany; Max Planck Institute of Molecular Plant Physiology, 14476 Potsdam-Golm, Germany; Max Planck Institute of Molecular Plant Physiology, 14476 Potsdam-Golm, Germany; Max Planck Institute of Molecular Plant Physiology, 14476 Potsdam-Golm, Germany; Max Planck Institute of Molecular Plant Physiology, 14476 Potsdam-Golm, Germany; Max Planck Institute of Molecular Plant Physiology, 14476 Potsdam-Golm, Germany

## Abstract

The cytochrome *b*_559_ heterodimer is a conserved component of photosystem II whose physiological role in photosynthetic electron transfer is enigmatic. A particularly puzzling aspect of cytochrome *b*_559_ has been its presence in etiolated seedlings, where photosystem II is absent. Whether or not the cytochrome has a specific function in etioplasts is unknown. Here, we have attempted to address the function of cytochrome *b*_559_ by generating transplastomic tobacco (*Nicotiana tabacum*) plants that overexpress *psbE* and *psbF*, the plastid genes encoding the 2 cytochrome *b*_559_ apoproteins. We show that strong overaccumulation of the PsbE apoprotein can be achieved in etioplasts by suitable manipulations of the promoter and the translation signals, while the cytochrome *b*_559_ level is only moderately elevated. The surplus PsbE protein causes striking ultrastructural alterations in etioplasts; most notably, it causes a condensed prolamellar body and a massive proliferation of prothylakoids, with multiple membrane layers coiled into spiral-like structures. Analysis of plastid lipids revealed that increased PsbE biosynthesis strongly stimulated plastid lipid biosynthesis, suggesting that membrane protein abundance controls prothylakoid membrane biogenesis. Our data provide evidence for a structural role of PsbE in prolamellar body formation and prothylakoid biogenesis and indicate that thylakoid membrane protein abundance regulates lipid biosynthesis in etioplasts.

## Teaser

Overexpression of a thylakoid protein in transplastomic plants that overexpress a subunit of a photosystem II component triggers massive membrane proliferation and stimulates lipid biosynthesis.

## Introduction

The thylakoid membranes of chloroplasts contain the protein complexes involved in the light reactions of photosynthesis. The linear electron transfer chain is composed of photosystem II (PSII), cytochrome *b*_6_*f* complex, and photosystem I (PSI). PSII catalyzes the first step in photosynthesis, in which electrons are extracted from water, thereby splitting water molecules into molecular oxygen and hydrogen ions. The electrons energized by light are ultimately used to reduce NADP+ to NADPH, and the proton gradient generated by water splitting and electron transfer is utilized to drive ATP synthesis by the chloroplast ATP synthase.

Advances in structural biology have provided us with high-resolution 3-dimensional structures of the thylakoid protein complexes that mediate the light reactions of photosynthesis (reviewed, e.g. in Nelson and Ben-Shem 2004; Nelson and Junge 2015). However, many important aspects of the function and the assembly of the complexes are still not or only insufficiently understood. The arguably most enigmatic constituent of PSII is the cytochrome *b*559 that has defied all attempts to elucidate its precise role in PSII function and biogenesis (Cramer and Zakharov 2022). Cytochrome *b*559 is present as a single heterodimer per PSII monomer and is composed of 2 short polypeptide chains (the α-subunit and the β-subunit) encoded by the genes *psbE* and *psbF*, respectively, in the plastid genome (Hird et al. 1986). The 2 polypeptides bind a heme that is ligated by 2 histidine residues, one from each subunit (Pakrasi et al. 1991; Stewart and Brudvig 1998; Morais et al. 2001). The 3-dimensional structure of PSII shows that cytochrome *b*559 is located in proximity to the reaction center subunit D2 but is spatially slightly separated from the major cofactors mediating electron transfer through the PSII complex (Shinopoulos and Brudvig 2012; Wei et al. 2016; van Bezouwen et al. 2017).

The presence of a heme in cytochrome *b*_559_ suggests a potential redox-related function in PSII, which, however, has been difficult to decipher. This is because the cytochrome also plays an essential role in the assembly of PSII (Müller and Eichacker 1999; Chu and Chiu 2016; Cramer and Zakharov 2022), and knockout of either the PsbE or the PsbF subunit results in loss of PSII complexes (Morais et al. 1998; Swiatek et al. 2003). Attempts to cleanly separate the structural role of cytochrome *b*559 in the PSII complex from its physiological function have met with limited success. Most mutants made so far by site-directed mutagenesis affected both PSII abundance and PSII function (Pakrasi et al. 1991; Bock et al. 1994; Morais et al. 2001). Proposed physiological functions of cytochrome *b*559 include participation in the oxidation of reduced plastoquinone (PQ) in the dark (Bondarava et al. 2003, 2010), a role in photoprotection of PSII (Shinopoulos and Brudvig 2012; Hamilton et al. 2014), and a function in cyclic electron flow within PSII (Stewart and Brudvig 1998; Shinopoulos and Brudvig 2012).

A peculiar feature of cytochrome *b*_559_ is its presence already in etioplasts, where no PSII accumulates (Blomqvist et al. 2008; Kanervo et al. 2008; Plöscher et al. 2009). While this could solely be related to the structural requirement for the cytochrome to facilitate the early steps of PSII assembly (Müller and Eichacker 1999; Chu and Chiu 2016), it could also indicate a specific function of cytochrome *b*559 in the dark. Here, we have attempted to provide an entry point into deciphering the function(s) of cytochrome *b*559 by overexpressing its subunits via manipulation of the expression of the small plastid operon that encodes the PsbE and PsbF proteins. We report that strong overexpression of the *psbE* operon is associated with a severe mutant phenotype in light-grown plants. The most striking consequence of *psbE* overexpression was a very strong increase in PsbE apoprotein accumulation in etioplasts, whereas only a moderate increase in cytochrome *b*559 content occurred. PsbE overaccumulation led to massive proliferation of prothylakoids and an endomembrane “snailing” phenotype in etioplasts of the transplastomic plants. Our results suggest that thylakoid membrane protein abundance in etioplasts regulates lipid biosynthesis and, in this way, controls prothylakoid biogenesis.

## Results

### Generation of transplastomic tobacco (*Nicotiana tabacum*) plants overexpressing the *psbEFLJ* operon

The 2 subunits of cytochrome *b*_559_ are encoded by the first 2 cistrons of the *psbEFLJ* operon. *psbE* encodes the α-subunit, and *psbF* encodes the β-subunit of the cytochrome *b*_559_ heterodimer (McNamara et al. 1997; Cramer and Zakharov 2022), and both genes are transcribed as part of a tetracistronic transcription unit that gives rise to a 1.1 kb RNA that comprises all 4 reading frames of the operon and does not undergo posttranscriptional cleavage into monocistronic mRNAs (Willey and Gray 1989; Bock et al. 1994). The 2 downstream genes of the operon, *psbL* and *psbJ*, encode small subunits of PSII that are required for stability and/or assembly of the photosystem (Hager et al. 2002; Swiatek et al. 2003) and affect electron transfer to PQ (Ohad et al. 2004).

**Figure 1.**
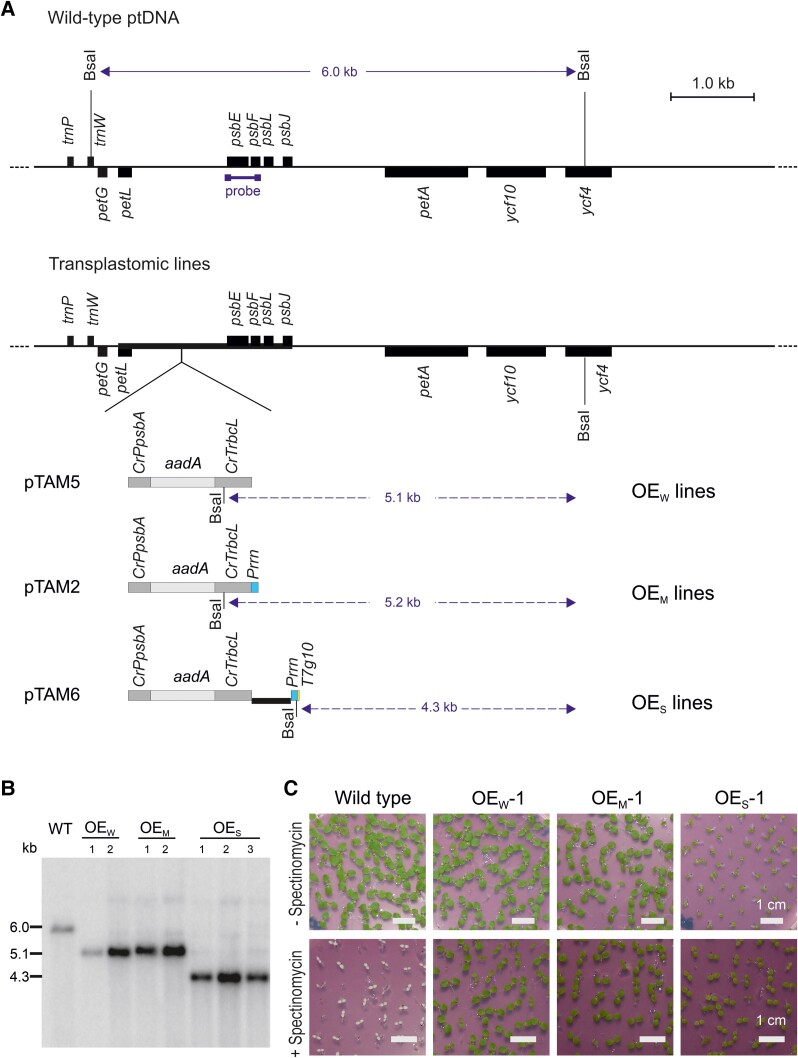
Generation of transplastomic tobacco lines designed to overexpress cytochrome *b*_559_. **A)** Physical map of the wild-type tobacco plastid genome (ptDNA) and the engineered plastid genomes in transplastomic lines generated with vectors pTAM5 (expected to confer weak overexpression of the *psbE* operon; OE_W_), pTAM2 (expected to yield intermediate overexpression lines; OE_M_), and pTAM6 (expected to result in strong *psbE* overexpression; OE_S_). Genes are represented as filled boxes and BsaI restriction sites used for RFLP analyses are indicated by thin vertical lines. The thick line marks the ptDNA region that is part of the pTAM plastid transformation vectors. The position of the hybridization probe and the length of the resulting RFLP fragments are also indicated. **B)** RFLP analysis of wild-type tobacco and the 3 sets of transplastomic lines (OE_W_, OE_M_, and OE_S_). Total plant DNA was digested with the restriction endonuclease BsaI (cf. **A**) and hybridized to the radiolabelled probe indicated in **A)**. All transplastomic lines are homoplasmic as evidenced by complete absence of the wild-type-specific band at 6.0 kb and exclusive presence of the band specific to the transformed plastid genomes (for fragment sizes in the 3 sets of transplastomic lines; see **A**). **C)** Inheritance assays demonstrating homoplasmy of transplastomic lines. Seeds were germinated in the absence or presence of spectinomycin, and seedlings were photographed 11 days after germination. Note the delayed growth of the OE_S_ seedlings, which is independent of the presence of spectinomycin. Uniform spectinomycin resistance of the progeny of all overexpression lines indicates their homoplasmic state and confirms maternal inheritance of the antibiotic resistance, as expected of a plastid-encoded trait. For tests of additional, independently generated transplastomic lines, see Supplementary Fig. S1.

To overexpress cytochrome *b*_559_, 3 constructs for chloroplast transformation in tobacco were designed. In the first construct, the selectable marker gene for plastid transformation, a chimeric *aadA* cassette conferring spectinomycin resistance (Svab and Maliga 1993), was inserted upstream *of the psbEFLJ* operon (pTAM5; Fig. 1A). Due to the absence of strict transcription termination mechanisms from chloroplasts (Stern and Gruissem 1987), expression of an upstream gene in sense orientation leads to read-through transcription (Zoubenko et al. 1994; Zhou et al. 2007; Lu et al. 2013) that causes overexpression of the downstream genes at the transcriptional and translational levels (Ghandour et al. 2023). The overexpression effects resulting from upstream marker insertion are usually mild (Ghandour et al. 2023), and we, therefore, preliminarily termed the transplastomic plants generated with vector pTAM5 “weak overexpressing lines” (OE_W_; Fig. 1A). Another design was based on additional insertion of a strong constitutive promoter, the ribosomal RNA operon promoter *Prrn*, downstream of the selectable marker gene cassette and upstream of the *psbEFLJ* operon (Fig. 1A). Due to its high transcriptional activity (Suzuki et al. 200*3*; Zhang et al. 2012; Caroca et al. 2013; Palomar et al. 2022), the *Prrn* promoter is expected to produce a substantial amount of additional *psbEFLJ* operon transcripts that could be translated to give rise to supernumerary cytochrome *b*559. The transplastomic lines generated with this overexpression construct (pTAM2) were preliminarily dubbed “medium overexpressing lines” (OE_M_; Fig. 1A). In both OE_M_ and OE_W_ plants, the level of overexpression depends on the extent to which increased transcript abundance leads to increased protein production (i.e. on the possible posttranscriptional regulation of *psbEFLJ* operon expression). As the regulation of *psbEFLJ* operon expression at the level of translation and protein stability is currently unknown, we designed an additional construct (pTAM6) to confer strong translational overexpression of at least the first cistron of the operon. This was achieved by placing the ribosomal RNA operon promoter *Prrn* upstream of the *psbEFLJ* operon and replacing the 5′ untranslated region (5′ UTR) of the *psbE* gene with the strong translation initiation signals from *gene10* of the bacteriophage T7 (*T7g10*; Kuroda and Maliga 2001; Fig. 1A). Due to the combination of a strong promoter with strong translation signals, this design was expected to yield strong overexpression lines (OE_S_; Fig. 1A).

The 3 constructs were transformed into tobacco plastids by stable chloroplast transformation using the biolistic protocol (Svab and Maliga 1993). Plastid-transformed (transplastomic) lines were selected by regeneration on spectinomycin-containing tissue culture medium, and primary antibiotic-resistant lines were subjected to additional rounds of regeneration in the presence of spectinomycin to eliminate residual wild-type copies of the highly polyploid plastid genome (Bock 2015; Greiner et al. 2020). Homoplasmy (i.e. absence of wild-type plastid DNA molecules) was verified by DNA gel blot analysis (Fig. 1B) and ultimately confirmed by inheritance assays that revealed a homogeneous population of antibiotic-resistant seedlings, consistent with the maternal inheritance of the plastid genome (Fig. 1C; Supplementary Fig. S1).

### Phenotype of transplastomic tobacco plants overexpressing the *psbEFLJ* operon

**Figure 2.**
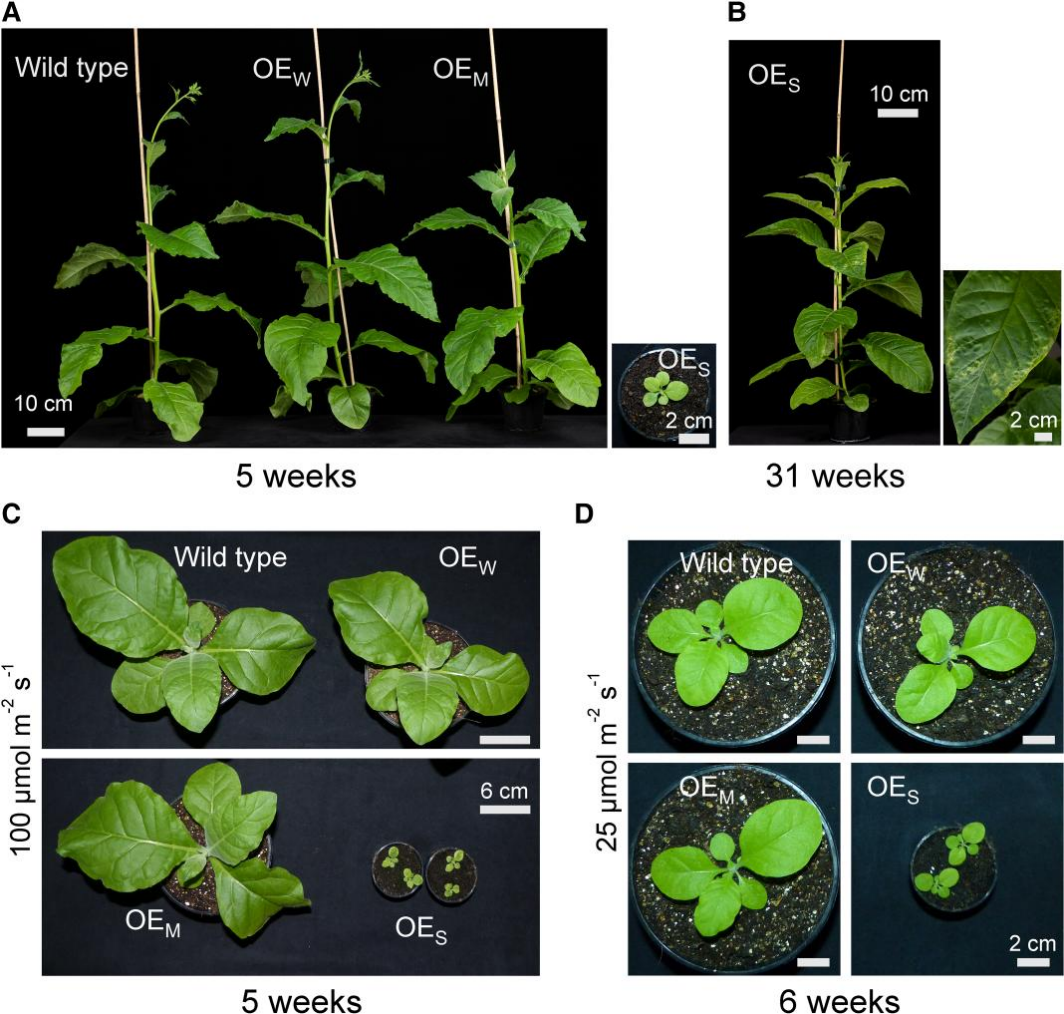
Phenotypes of transplastomic plants generated in this study. **A)** Visible phenotypes of wild type, OE_W_, OE_M_, and OE_S_ plants grown under standard greenhouse conditions (wild type, OE_W_, and OE_M_) or nursery conditions (OE_S_). All plants were raised under nursery conditions (see Materials and methods) for 1 month and photographed 3 weeks after transfer of the wild type, OE_W_, and OE_M_ plants to the greenhouse. OE_S_ plants remained under nursery conditions, due to their drastic growth retardation. **B)** Phenotype of an OE_S_ plant 31 weeks after germination, when it had reached a similar developmental stage as the wild type, OE_W_, and OE_M_ plants shown in **A)**. A typical leaf exhibiting necrotic sectors is shown at the right. **C)** Phenotypes of wild type, OE_W_, OE_M_, and OE_S_ plants grown at 100 *µ*mol photons m^−2^ s^−1^ light intensity for 5 weeks. **D)** Plant phenotypes upon growth at 25 *µ*mol photons m^−2^ s^−1^ for 6 weeks. Note the overall delayed growth of wild type, OE_W_, and OE_M_ plants but also the reduced growth difference to the OE_S_ plants under low-light conditions.

Having obtained homoplasmic transplastomic lines for all 3 overexpression constructs, we next investigated the phenotypes of the lines by growing them from seeds along with the wild type under a variety of growth conditions. When grown under standard greenhouse conditions (average light intensity: 260 *µ*mol photons m^−2^ s^−1^), OE_W_ plants were indistinguishable from wild-type plants. OE_M_ plants displayed a slight growth retardation (Fig. 2A), whereas OE_S_ plants were pale green and severely delayed in growth and development. Interestingly, the leaves of OE_S_ plants developed necrotic sectors (Fig. 2B; Supplementary Fig. S2A), possibly suggesting that the plants suffer from severe photooxidative damage that leads to cell death induced by reactive oxygen species (ROS).

When grown under low-light conditions (100 *µ*mol photons m^−2^ s^−1^), the mutant phenotype of the OE_S_ plants was less severe (Fig. 2C). Under extreme low-light conditions (25 *µ*mol photons m^−2^ s^−1^), the growth difference between the wild type and the OE_S_ mutants diminished further (Fig. 2D), and no tissue necroses were seen, indicating that cell death under higher light intensities was indeed triggered by photooxidative stress.

Next, we measured a set of photosynthesis-related parameters in the youngest fully expanded leaves of wild-type plants and 2 to 3 independent lines of each OE mutant grown in a controlled environment chamber with 300 *µ*mol photons m^−2^ s^−1^ light intensity, similar to our standard greenhouse conditions (Table 1). Due to their delayed development, the OE_S_ mutants were grown for an extra week prior to the measurements. Analysis of chlorophyll contents confirmed the visual pigment-deficient phenotype of the OE_S_ mutants. Also, the maximum quantum efficiency of PSII in the dark-adapted state (F_V_/F_M_) was clearly reduced in OE_S_ plants (Table 1), an effect that was mainly attributable to increased minimum chlorophyll-*a* fluorescence emission F_0_ in the OE_S_ plants (Table 1).

**Table 1.** Quantification of chlorophyll a/b ratio, chlorophyll content per leaf area, the maximum quantum efficiency of PSII in the dark-adapted state (F_V_/F_M_), and the amount of photosynthetic complexes in thylakoid membranes of wild type, OE_W_, OE_M_, and OE_S_ plants

Parameter	Wild type	OE_W_-1	OE_W_-2	OE_M_-1	OE_M_-2	OE_S_-1	OE_S_-2	OE_S_-3
Chlorophyll a/b	**4.25** ± 0.10	**4.13** ± 0.13	**4.11** ± 0.04	**4.05** ± 0.04	**3.98** ± 0.07^a^	**3.98** ± 0.19^a^	**4.03** ± 0.17	**4.03** ± 0.16
Chlorophyll (mg m^−2^)	**484.7** ± 104.7	**488.2** ± 64.9	**487.2** ± 64.2	**507.2** ± 72.3	**552.3** ± 94.5	**111.8** ± 12.9^c^	**104.9** ± 22.9^c^	**124.6** ± 27.2^c^
F_0_	**0.25** ± 0.02	**0.25** ± 0.02	**0.25** ± 0.02	**0.25** ± 0.02	**0.27** ± 0.02	**0.34** ± 0.04^a^	**0.36** ± 0.07^a^	**0.36** ± 0.06^a^
F_V_/F_M_	**0.81** ± 0.00	**0.81** ± 0.01	**0.80** ± 0.00	**0.80** ± 0.01	**0.79** ± 0.02	**0.62** ± 0.05^c^	**0.58** ± 0.08^c^	**0.60** ± 0.07^c^
PSII (mmol mol Chl.^−1^)	**3.12** ± 0.25	**3.17** ± 0.49	**3.20** ± 0.33	**2.70** ± 0.12	**2.73** ± 0.17	**4.73** ± 1.11^b^	**4.84** ± 0.94^b^	**3.92** ± 0.40
Cyt-bf (mmol mol Chl.^−1^)	**1.34** ± 0.08	**1.35** ± 0.26	**1.21** ± 0.10	**0.73** ± 0.11^c^	**0.78** ± 0.12^c^	**0.61** ± 0.11^c^	**0.63** ± 0.06^c^	**0.50** ± 0.07^c^
PSI (mmol mol Chl.^−1^)	**2.43** ± 0.07	**2.36** ± 0.10	**2.36** ± 0.08	**2.42** ± 0.06	**2.40** ± 0.03	**2.00** ± 0.20^c^	**2.05** ± 0.15^c^	**2.09** ± 0.25^c^
PSII (µmol m^−2^)	**1.50** ± 0.27	**1.55** ± 0.31	**1.54** ± 0.11	**1.36** ± 0.15	**1.50** ± 0.26	**0.52** ± 0.10^c^	**0.49** ± 0.09^c^	**0.49** ± 0.12^c^
Cyt *b*_6_*f* (µmol m^−2^)	**0.65** ± 0.15	**0.66** ± 0.15	**0.59** ± 0.11	**0.37** ± 0.04^c^	**0.43** ± 0.09^c^	**0.07** ± 0.02^c^	**0.07** ± 0.01^c^	**0.06** ± 0.01^c^
PSI (µmol m^−2^)	**1.18** ± 0.27	**1.15** ± 0.18	**1.15** ± 0.15	**1.23** ± 0.17	**1.33** ± 0.23	**0.22** ± 0.02^c^	**0.22** ± 0.04^c^	**0.26** ± 0.03^c^
Cyt *b_559_*/etioplast	**5.032 × 10^6^**	…	…	…	…	**7.248 × 10^6^**		…
Cyt *b_6_f*/etioplast	**1.199 × 10^6^**	…	…	…	…	**0.066 × 10^6^**		…

The chlorophyll a/b ratio, the chlorophyll content per leaf area, the minimum chlorophyll-*a* fluorescence emission in the dark-adapted state (F_0_), and the maximum quantum efficiency of PSII in the dark-adapted state (F_V_/F_M_) were measured (*n* = 12; see Materials and methods). The contents of photosynthetic complexes (PSII, cytochrome *b*_6_*f* complex, and PSI) were determined using isolated thylakoids and normalized on a chlorophyll basis (mmol mol Chl^−1^) or leaf area basis (µmol m^−2^; *n* = 6). Contents of cytochrome *b*_559_ and cytochrome *b*_6_*f* complex per etioplast were determined in etioplasts isolated from the wild type and the OE_S_ mutants (OE_S_-1 and OE_S_-2). Average values are given in bold, followed by the standard deviation. Due to the large amount of plant material needed for etioplast isolation, no biological replicates could be performed for this data set, thus precluding statistical analysis. Etioplasts isolated from OE_S_-1 and OE_S_-2 plants were pooled for the measurement.

^a^Values significantly different only to the wild type.

^b^Significant differences between OE_S_ and OE_M_.

^c^All lines of the construct significantly different to all other genotypes (*P* < 0.05; ANOVA, pairwise multiple comparison procedure analyses [Holm–Sidak method] in SigmaPlot version 14.5).

To better understand the increased susceptibility of the OE_S_ mutants to photooxidative damage, we measured light response curves of chlorophyll-*a* fluorescence parameters (Supplementary Fig. S3). Light response curves of photoprotective nonphotochemical quenching (NPQ) were indistinguishable from wild type in the 2 independent OE_W_ mutants, whereas both the 2 independent OE_M_ mutants and the 3 OE_S_ mutants showed impaired NPQ (Supplementary Fig. S3A). In OE_M_ plants, NPQ was similar to that in the wild type under low light up to the growth light intensity (indicated by the dashed line in Supplementary Fig. S3A), but failed to be fully induced at higher light intensities. OE_S_ plants showed a clear induction of NPQ already in low light, but failed to further activate NPQ to wild-type levels at higher light intensities, similar to OE_M_ plants. Light response curves of qL, a measure of the redox state of the PSII acceptor side (Kramer et al. 2004), revealed that, with increasing light intensity, the PSII acceptor side became increasingly reduced in the wild type and the OE_W_ plants (Supplementary Fig. S3B). Notably, in OE_M_ and especially in OE_S_ plants, the reduction of the PSII acceptor side occurred at much lower light intensities than in the wild type, pointing to an inefficient electron transfer from PSII via the cytochrome *b_6_f* complex to PSI. Finally, the light response curve of the nonregulated dissipation of excitation energy in PSII, Y(NO), was determined (Supplementary Fig. S3C). In line with their impaired NPQ induction under high light, and their more reduced PSII acceptor side, OE_M_ plants suffered from increased Y(NO) especially at light intensities exceeding the growth light intensity of 300 *µ*mol photons m^−2^ s^−1^. By contrast, OE_S_ showed clearly increased Y(NO) already in low light. Taken together, the impaired NPQ, the increased reduction of the PSII acceptor side, and the strongly elevated Y(NO) point to impaired linear electron transport and increased sensitivity of PSII to photoinhibition especially in the OE_S_ mutants and likely explain their bleached and necrotic leaf phenotype.

Spectroscopic quantification of photosynthetic complexes in the thylakoid membrane revealed strongly reduced levels of all complexes (normalized to leaf area; Table 1) in OE_S_ plants. Both OE_S_ and OE_M_ plants showed reduced levels of the cytochrome *b*_6_*f* complex, a phenomenon that has been reported previously in transplastomic plants harboring the *aadA* selectable marker cassette upstream of and in antisense orientation to the *petA* gene (Fig. 1A; Loiacono et al. 2019; Ghandour et al. 2023). Transcriptional read-through from the strong promoter driving the *aadA* generates antisense transcripts to *petA*, *e*ncoding cytochrome *f*, and leads to reduced cytochrome *b*_6_*f* complex biogenesis, but in previously analyzed transplastomic lines, did not cause a growth phenotype under standard conditions (Ghandour et al. 2023). Also, decreased accumulation of the cytochrome *b*_6_*f* complex was previously shown to impair linear electron transport and thylakoid membrane energization and prevent normal induction of NPQ (Loiacono et al. 2019, 2022), as observed here for both OEM and OES plants.

In OE_S_ plants, besides the decreased cytochrome *b_6_f* complex accumulation, PSI accumulation was reduced to only 20% of wild-type levels (on a per-leaf-area basis), while PSII accumulation was less affected. To assess the impact of the altered photosynthetic complex accumulation on antenna distribution between both photosystems, chlorophyll-*a* fluorescence emission spectra at 77 K were measured (Supplementary Fig. S4). All spectra were normalized to the emission maximum of PSII at 685 nm wavelength. Despite reduced accumulation of PSII and strong PSII photoinhibition in OE_S_ plants, no evidence for the presence of uncoupled LHCII (which show emission maxima at wavelengths below 685 nm) was obtained. While the emission spectra were similar for the wild type and the OE_W_ and OE_M_ mutants, the PSI emission maximum was shifted from 733 nm (in the wild type and the OE_W_ and OE_M_ plants) to shorter wavelengths in the OE_S_ plants, with a pronounced increase in emission between 725 and 710 nm. This is indicative of the presence of uncoupled LHCI antenna proteins, as previously reported for numerous mutants affected in PSI assembly and/or accumulation (Schöttler et al. 2007; Albus et al. 2010; Krech et al. 2012; Rolo et al. 2024). Uncoupled LHCI could further contribute to the increased F_0_ and decreased F_V_/F_M_ of the OE_S_ mutant plants.

### Analysis of *psbE* operon overexpression

**Figure 3.**
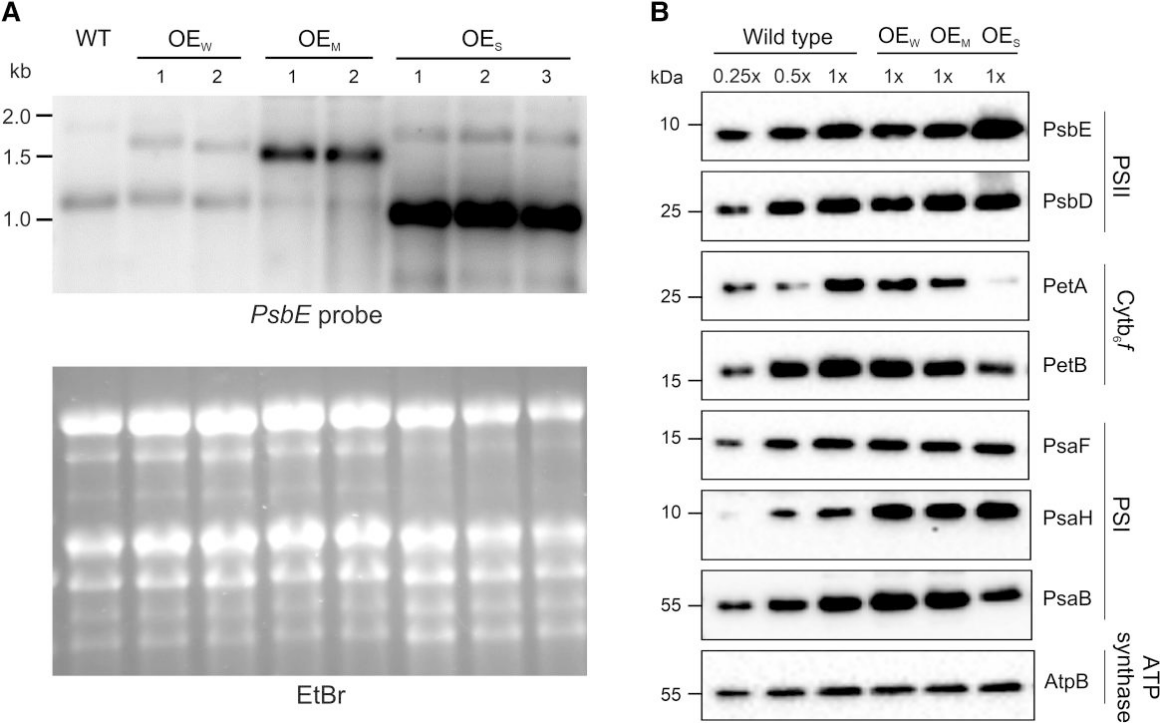
Analysis of *psbE* operon transcripts and thylakoid protein accumulation in OE_W_, OE_M_, and OE_S_ transplastomic tobacco plants. **A)** Northern RNA blot analysis of transcripts from the *psbE-psbF-psbL-psbJ* operon. Samples of 6.0 *µ*g total cellular RNAs were electrophoretically separated in a 1% denaturing agarose gel and hybridized to a radiolabeled *psbE* probe generated by PCR amplification with gene-specific primers (Supplementary Table S1). Sizes of relevant RNA marker bands are given in kb. Transcription from the native *psbE* operon promoter produces a 1.1 kb tetracistronic RNA in the wild type that is also present in the OE_W_ and OE_M_ transplastomic plants. The OE_W_ lines display an additional larger transcript resulting from read-through transcription from the upstream *aadA* marker gene. The OE_M_ lines express an abundant larger transcript of ∼1.4 kb that originates from the strong *Prrn* promoter placed upstream of the native *psbE* operon promoter. The OE_S_ transplastomic lines harbor the *Prrn* promoter downstream of the native *psbE* operon promoter and, therefore, produce a highly abundant transcript of similar size as the wild type (cf. Fig. 1A). The ethidium bromide (EtBr)–stained agarose gel prior to blotting is shown to confirm equal RNA loading. **B)** Immunoblot analysis of diagnostic components of the major photosynthetic complexes residing in thylakoid membranes. Samples of 20 *µ*g total cellular protein were separated by electrophoresis in 12% SDS-PAA (polyacrylamide) gels, blotted, and immunodecorated with antibodies against the subunits indicated at the right. To facilitate semiquantitative assessment of protein accumulation, a dilution series (1/4, 1/2 and 1×) of the wild-type sample was included.

To assess the level of overexpression in OE_W_, OE_M_, and OE_S_ plants at the mRNA level, RNA gel blot analyses were conducted. As expected, *aadA* marker insertion upstream of the *psbE* operon led only to mildly increased transcript levels. This was evidenced by largely unaltered presence of the 1.1 kb *psbE* operon transcript and presence of the additional read-through transcript initiating from the upstream *aadA* and its strong promoter (Figs. 1A and 3A). Integration of the additional ribosomal RNA operon promoter (*Prrn*) upstream of the *psbE* operon in OE_M_ plants led to the expected strong increase in transcript accumulation compared to the wild type (Fig. 3A). *Prrn* insertion immediately upstream of the *psbE* coding region (and replacement of the endogenous *psbE* 5′UTR with the T7 *gene10* leader sequence) led to an even stronger overaccumulation of *psbE* operon transcripts in the OE_S_ mutant plants (Figs. 1A and 3A).

The consequences of *psbE* operon overexpression at the level of protein complex accumulation in the thylakoid membrane were evaluated by a set of immunoblot analyses using antibodies against diagnostic subunits of all complexes. To facilitate semiquantitative assessment, a dilution series of the wild-type sample was included in all blots. These experiments revealed slightly increased levels of PsbE (the α-subunit of cytochrome *b*_559_), but largely similar levels of the 2 photosystems and the ATP synthase in all transplastomic lines (Fig. 3B). It should be noted that the protein gels were loaded based on equal amounts of total cellular protein, whereas our spectroscopic measurements were normalized to leaf area or chlorophyll content. Thus, the strongly reduced chlorophyll contents in the OE_S_ mutant plants (Supplementary Fig. S2B) explain the apparent discrepancies between the complex accumulation levels determined by immunoblotting (Fig. 3B) and the decreased complex accumulation levels measured spectroscopically (for a direct quantitative comparison of complex accumulation per mole chlorophyll and per square meter leaf area, see Table 1).

The immunoblot data also confirmed the reduced accumulation of the cytochrome *b*_6_*f* complex that had been determined in our spectroscopic quantification of thylakoid protein complexes (Table 1; Fig. 3B) and also had been seen in previously generated transplastomic lines that harbored the selectable marker gene in the same region of the plastid genome (Loiacono et al. 2019; Ghandour et al. 2023). To confirm that antisense transcription into the *petA* gene (encoding cytochrome *f*) is causally responsible for this effect, *petA* transcript levels were assessed by RNA gel blot analysis (Supplementary Fig. S5). The data confirmed the antisense RNA effect and showed reduced levels of *petA* mRNA in all transplastomic lines, with the reduction being particularly pronounced in the OE_S_ mutant plants (Supplementary Fig. S5).

### Strong overaccumulation of the PsbE protein in etioplasts of OE_S_ mutant plants

The disturbed photosynthesis and the massive photooxidative damage in the OE_S_ mutant limit the insights that can be gained from analyses of light-grown plants. For this reason, and because a key motivation for generating the transplastomic overexpression plants was to understand the biological role of cytochrome *b*_559_ in etioplasts (where no PSII accumulates; Blomqvist et al. 2008; Kanervo et al. 2008; Plöscher et al. 2009), we focused our subsequent analyses on etiolated material. In the absence of light, secondary effects from photooxidative damage are excluded, thus facilitating the elucidation of the primary effects of altered cytochrome *b*559 accumulation levels.

**Figure 4.**
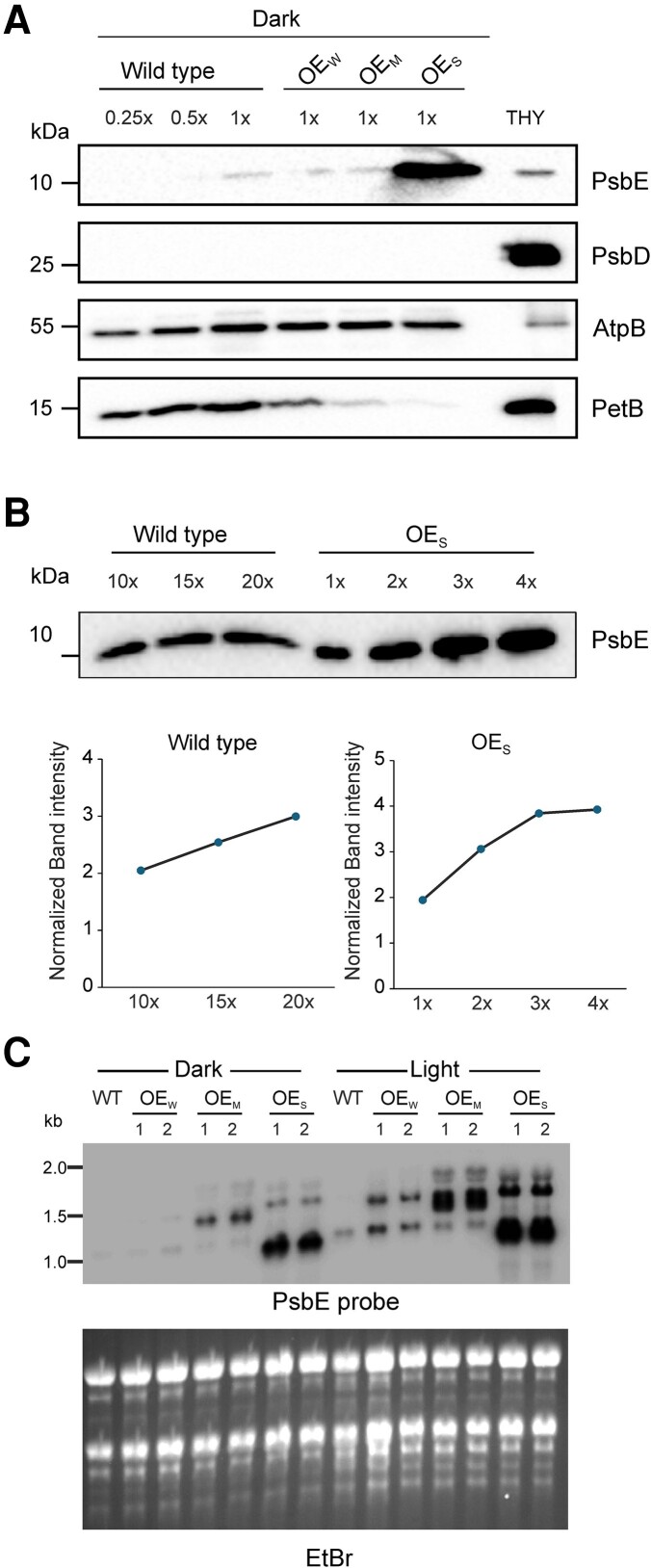
Overexpression of *psbE* at the protein and mRNA levels. **A)** Massive overaccumulation of PsbE in etioplasts, as shown by immunoblot analysis of diagnostic subunits of photosynthetic complexes residing in thylakoid membranes. Samples of 20 *µ*g total protein extracted from etiolated seedlings of the wild type and the cytochrome *b*_559_ overexpression plants were separated by 12% SDS-PAGE (polyacrylamide gel electrophoresis), blotted, and immunodecorated with antibodies against the diagnostic protein subunit denoted at the right. To allow for semiquantitative assessment, a dilution series (0.25×, 0.5×, and 1×) of the wild-type sample was included in each blot. Thylakoids extracted from 7-day-old tobacco seedlings (THY; equivalent to 70 ng chlorophyll) were loaded as an additional control. Note the accumulation of subunits of the cytochrome *b*_6_*f* complex and the ATP synthase, known to be present already in etioplasts, but absence of PSII complexes (evidenced by the essential subunit PsbD) that accumulate in a light-dependent manner. **B)** Semiquantitatively assessment of the level of overaccumulation of the PsbE protein in etiolated OE_S_ seedlings. Dilution series of the wild-type sample (10×, 15×, and 20×), and the OE_S_ sample (1×, 2×, 3×, and 4×) were analyzed by immunoblotting. Note the comparable PsbE hybridization signal intensities in 1× OE_S_ and 10× wild type. The experiment was performed twice and a representative blot is shown. The graphs show the quantification of the signal intensities for each band in the blot with the ImageJ software. Note linearity over the entire range in the wild-type samples and linearity between 1× and 3× in the OE_S_ samples. **C)** RNA gel blot analyses showing the accumulation of *psbE* operon transcripts in etiolated and light-exposed (48 h post illumination) seedlings. Samples of 3 *µ*g total RNA from each genotype were analyzed. The 1.1 kb tetracistronic *psbE* operon transcript (cf. Fig. 3A) is the major RNA species accumulating in etiolated OE_S_ seedlings. The ethidium bromide–stained agarose gel prior to blotting is shown below the blot (EtBr).

To assess cytochrome *b*_559_ accumulation, we spectroscopically determined its amount in intact etioplasts isolated from the wild type and the OE_S_ mutants. Per etioplast, a 1.44-fold increase of cytochrome *b*_559_ was detected in OE_S_ plants compared to the wild type (Table 1). Next, immunoblots with antibodies against the PsbE protein were conducted using total protein extracted from etiolated seedlings. While PsbE accumulation levels in OE_W_ and OE_M_ plants were similar to those in the wild type, the OE_S_ plants showed very strong overaccumulation of PsbE (Fig. 4A). Control blots with antibodies against the essential PSII subunit PsbD, the ATP synthase subunit AtpB, and the cytochrome *b*_6_*f* complex subunit PetB confirmed the absence of PSII complexes from etioplasts and the presence of the ATP synthase and cytochrome *b*_6_*f* complex subunits. Accumulation of the latter was decreased in OE_S_, similar to the situation in green tissue (Table 1), and in line with the reduced cytochrome *b*_6_*f* complex accumulation in etioplasts of the OE_S_ mutants (Table 1). Assessment of the intensity of the PsbE signals in comparison to a dilution series from wild-type samples suggested that the OE_S_ etioplasts accumulate approximately 10-fold more PsbE protein than wild-type etioplasts (Fig. 4B). Strikingly, we noticed a much more pronounced increase in PsbE protein accumulation in etioplasts than in green tissue of the OE_S_ plants (Figs. 3B and 4A), although the *psbE* operon transcript abundance was less increased in etiolated OE_S_ seedlings than in light-exposed seedlings (Fig. 4C). Whether or not this difference is due to unassembled PsbE apoprotein being efficiently degraded in chloroplasts (but not in etioplasts) remains to be determined.

### Ultrastructural changes in etioplasts of the OE_S_ mutant

**Figure 5.**
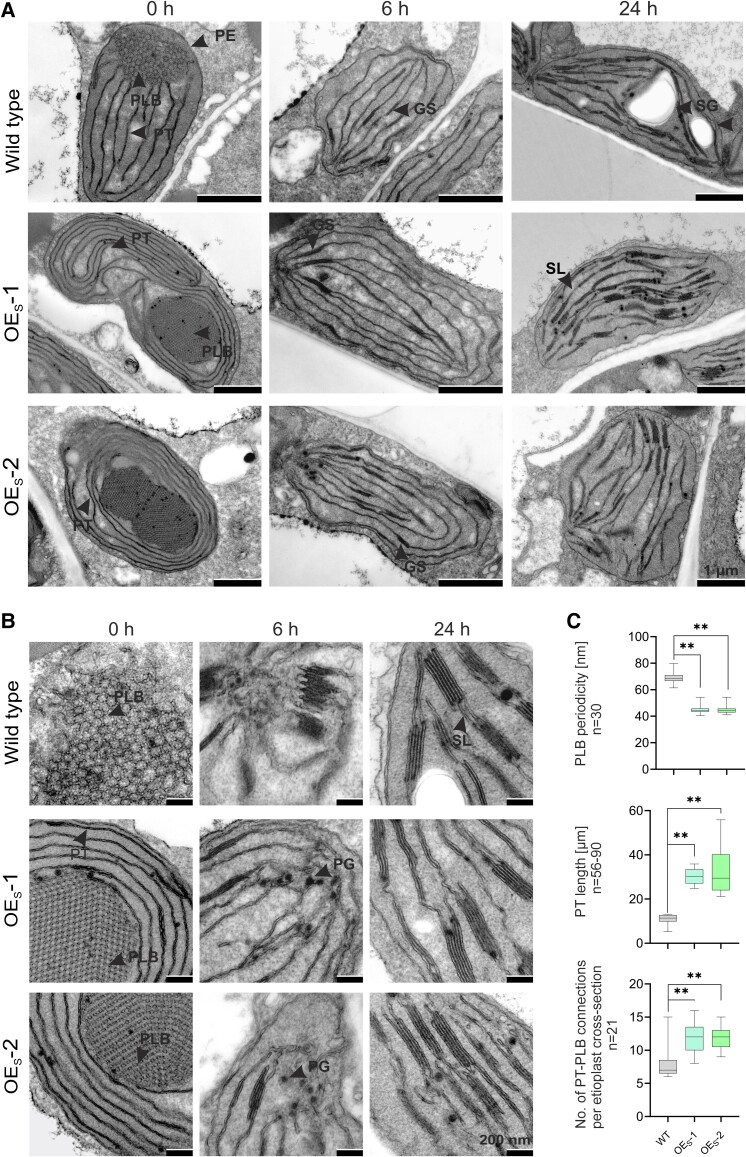
Strong cytochrome *b*_559_ overexpression plants display condensed PLBs and membrane overproliferation in etioplasts. **A)** Representative TEM images of etioplasts in cotyledons of seedlings grown in the dark (0 h) and at 2 different time points of deetiolation (6 and 24 h). PLB, prolamellar body; PT, prothylakoid; PE, plastid envelope; GS, grana stack; SG, starch grain; SL, stroma lamella; PG, plastoglobule. Scar bar: 1 *µ*m. **B)** PLB and membrane ultrastructure at larger magnification. Scar bar: 200 nm. **C)** Quantification of ultrastructural traits in etioplasts. Values represent means ± maxima and minima of the replicates of each genotype. For quantification of PT length in the wild type, 90 PTs from 10 etioplasts were measured. For the 2 independently generated transplastomic OE_S_ (strong overexpressing) lines, OE_S_-1 and OE_S_-2, 67 and 56 PTs, respectively, from 10 etioplasts each were measured. Centerlines define the median, box limits mark the upper and lower quartiles, and whiskers indicate the 1.5× interquartile range. Asterisks indicate significant differences among mean values of wild type, OE_M_ (medium overexpressing) and OE_S_ samples (1-way ANOVA with Tukey's post hoc honestly significant difference [HSD] test; **P < 0.01).

In order to examine the structural consequences of the strong overaccumulation of PsbE protein in etioplasts of transplastomic OE_S_ seedlings, transmission electron microscopy (TEM) was undertaken. These analyses revealed striking alterations of etioplast ultrastructure in the OE_S_ mutant plants (Fig. 5A). While wild-type etioplasts contained a prolamellar body (PLB) with a few prothylakoids emerging from it, mutant etioplasts showed an extreme proliferation of prothylakoids, with multiple layers of membranes being coiled up and surrounding the PLB—a phenotype we refer to as “prothylakoid snailing” (Fig. 5A; Supplementary Fig. S6).

To follow the changes in plastid ultrastructure during deetiolation, we also investigated seedlings after 6 and 24 h of illumination. Remarkably, already after 6 h in the light, the snailing phenotype had vanished, the coiled-up prothylakoids had disappeared, and the developing chloroplasts were nearly indistinguishable from those in deetiolating wild-type seedlings (Fig. 5A).

Analysis of TEM images at higher resolution (Fig. 5B) revealed that the ultrastructure of the PLB was also altered in etioplasts of the OE_S_ mutant (Fig. 5B; Kowalewska et al. 2016; Sandoval-Ibánez et al. 2021). The paracrystalline ordering in the lattice structure showed a significantly reduced periodicity in the mutant seedlings (Fig. 5, B and C). In addition, the number and length of prothylakoids connecting to the PLB was increased in the OE_S_ mutants (Fig. 5). Taken together, these observations demonstrate that overaccumulation of PsbE affects both prothylakoid biogenesis and PLB structure in etioplasts.

### Delayed greening and induction of ROS marker genes in OE_S_ mutant plants

**Figure 6.**
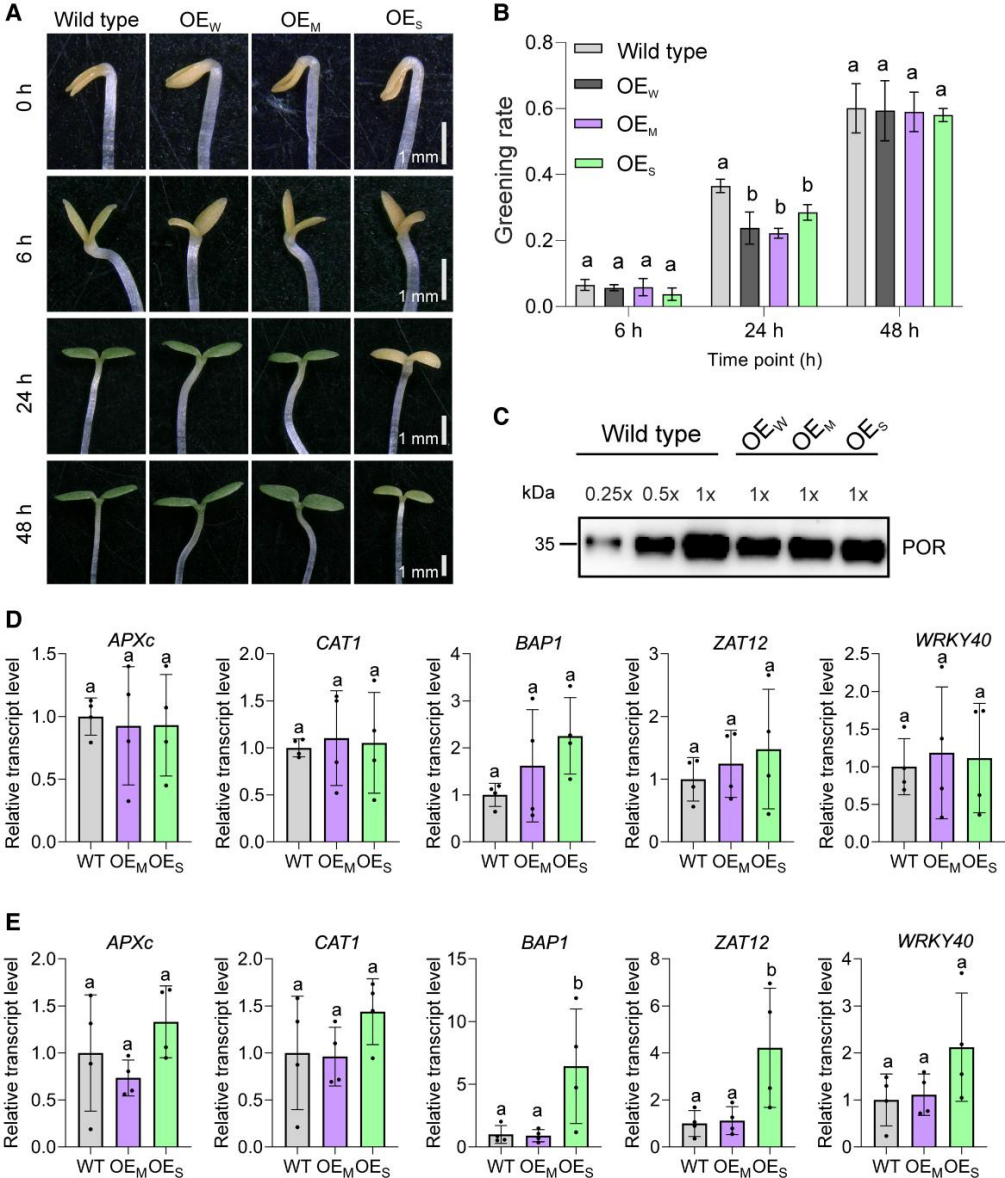
Strong cytochrome *b*_559_ overexpression is linked to photooxidative stress upon deetiolation. **A)** Representative images of cotyledons from wild-type seedlings and transplastomic OE_W_, OE_M_, and OE_S_ seedlings during deetiolation. The seedlings were germinated and initially grown in the dark for 7 days and then transferred to the light. Pictures were taken at different time points of light exposure as indicated at the left. **B)** Greening rate of seedlings during deetiolation. Greening rates were measured as the chlorophyll content (µg chlorophyll/mg fresh weight) at a given time point relative to the chlorophyll content of completely greened plants 4 days after light exposure (in percent). Values represent means ± Sd (standard deviation) of at least 3 independent biological replicates. Lowercase letters indicate statistically significant differences between mean values of each genotype (*P* < 0.05, 1-way ANOVA with Tukey's post hoc honestly significant difference [HSD] test). **C)** Immunoblot analysis of POR protein accumulation in etiolated seedlings of the wild type and the transplastomic cytochrome *b*_559_ overexpression lines. Samples of 20 *µ*g extracted total protein were separated by 12% SDS-PAGE (polyacrylamide gel electrophoresis), blotted, and immunodecorated with antibodies against POR (Supplementary Table S2). To facilitate semiquantitative assessment, a dilution series (0.25×, 0.5×, and 1×) of the wild-type sample was included. **D** and **E)** Relative expression levels of selected ROS-responsive genes in OE_M_ and OE_S_ seedlings after light exposure for 5 min **D)** and 30 min **E)** compared to the wild type. Expression levels were measured by RT-qPCR using primers for the H_2_O_2_ marker genes *APXc* and *CAT1*, the ^1^O_2_ marker gene *BAP1*, and the ROS-responsive transcription factors *ZAT12* and *WRKY40*. *ACTIN* was used as an internal control. Values represent means ± Sd (standard deviation) of 4 independent biological replicates. Lowercase letters indicate statistically significant differences between mean values among different genotypes (*P* < 0.05, 1-way ANOVA with Tukey's post hoc honestly significant difference [HSD] test).

In order to investigate the possible physiological importance of PsbE levels in etioplasts, deetiolation experiments were conducted, and the speed of greening was assessed both visually (Fig. 6A) and by following the increase in chlorophyll accumulation over time (Fig. 6B; Supplementary Fig. S7). While etiolated OE_W_ and OE_M_ seedlings displayed a similar rate of greening as the wild type (in that seedlings were visibly green after 24 h), the greening of the OE_S_ mutant was clearly delayed and seedlings were still largely yellowish after 24 h of illumination (Fig. 6A). The rate of chlorophyll accumulation relative to the fully greened state of each line showed no significant differences between the 3 transplastomic lines (and all of them showed a slight delay compared to the wild type; Fig. 6B, 24 h time point), but the absolute rate of chlorophyll accumulation was significantly slower in the OE_S_ mutant than in the other 2 mutants (Supplementary Fig. S7). This difference between relative and absolute chlorophyll accumulation rates is largely due to the lower chlorophyll content of fully deetiolated OE_S_ seedlings (cf. Table 1 and Supplementary Fig. S2).

Determination of the accumulation levels of NADPH:protochlorophyllide oxidoreductase (POR), the most abundant etioplast protein (that catalyzes the light-dependent conversion of protochlorophyllide to chlorophyllide a and has been identified as a key structural determinant of the PLB; Floris and Kühlbrandt 2021), revealed no pronounced difference in POR protein levels (relative to total seedling protein) in the 3 transplastomic mutants, indicating that the massive overaccumulation of PsbE does not occur at the expense of other major etioplast proteins (Fig. 6C).

To gain insights in the early processes during deetiolation, we measured the expression levels of selected ROS-responsive genes in OE_M_ and OE_S_ seedlings after illumination for 5 and 30 min, respectively (Fig. 6, D and E). By RT-qPCR, we analyzed the H_2_O_2_ marker genes *APXc* and *CAT1*, the ^1^O_2_ marker gene *BAP1*, and the ROS-responsive transcription factors *ZAT12* and *WRKY40*. After 30 min of light exposure, the expression of the ^1^O_2_ marker *BAP1* and the transcription factor *ZAT12* was more strongly induced in the OE_S_ seedlings than in the wild type and the OE_M_ seedlings (Fig. 6, D and E). By contrast, H_2_O_2_ marker genes did not show a similar upregulation. Thus, increased ^1^O_2_ generation at PSII in OE_S_ seedlings during the deetiolation process likely makes seedlings light sensitive and prone to photooxidative stress and may also explain the necrotic cell death observed in mature OE_S_ plants (Fig. 2B; Supplementary Fig. S2A; Kim and Apel 2013).

### PsbE overaccumulation and prothylakoid snailing are accompanied by changes in plastid lipid accumulation

The observed prothylakoid snailing phenotype in etioplasts of OE_S_ seedlings (Fig. 5A; Supplementary Fig. S6) raises the question of how the enormous membrane proliferation is supported by appropriate lipid supply. We, therefore, set out to determine how membrane lipid biosynthesis responds to the presence of increased amounts of prothylakoid proteins in etiolated OE_S_ seedlings. To this end, lipid species were identified in etiolated seedlings of the wild type and the OE_M_ and OE_S_ transplastomic lines by a nontargeted UPLC-MS-based lipidomic approach (Armarego-Marriott et al. 2019). The major lipid classes contributing to the formation of (pro)thylakoid membranes and PLBs, monogalactosyldiacylglycerol (MGDG), digalactosyldiacylglycerol (DGDG), sulfoquinovosyldiacylglycerol (SQDG), and phosphatidylglycerol (PG) (Ohlrogge and Browse 1995; Li et al. 2016), were quantified as sum of all respective diacylglyceride (DAG) species per fresh weight (Supplementary Data Set 1). These 4 lipid classes remained largely unchanged in etiolated OEM seedlings but were significantly increased in etiolated OE_S_ seedlings compared to the wild type, by factors of 1.15 (sum of DGDGs; heteroscedastic *t* test *P* = 0.0297), 1.29 (sum of MGDGs; heteroscedastic *t* test, *P* = 0.0034; Supplementary Data Set 1), 1.22 (sum of PG; heteroscedastic *t* test, *P* = 0.00085), and 1.26 (sum of SQDG; heteroscedastic *t* test, *P* = 0.00291). These observations confirm the TEM results and associate the prothylakoid snailing phenotype in OE_S_ seedlings to increased lipid accumulation (rather than lipid rearrangement).

The galactolipids MGDG and DGDG together can account for up to 80% of the total thylakoid lipids (Boudière et al. 2014). Tobacco, like Arabidopsis (*Arabidopsis thaliana*), is a 16:3 plant that operates 2 biosynthesis pathways to synthesize plastid lipids (Koiwai et al. 1981; Mongrand et al. 1998; Hölzl and Dörmann *201*9). The prokaryotic pathway uses de novo synthesized fatty acid within plastids for lipid biosynthesis, whereas the eukaryotic pathway reimports DAG moieties that were synthesized in the endoplasmic reticulum (ER) and are then delivered to the plastid envelope (Hölzl and Dörmann 2019). The prokaryotic pathway generates plastid lipids with 16-C fatty acids at the sn-2 position, whereas the eukaryotic pathway produces lipids with an 18-C fatty acid at the sn-2 position (Ohlrogge and Browse 1995; Li et al. 2016; Hölzl and Dörmann 2019).

**Figure 7.**
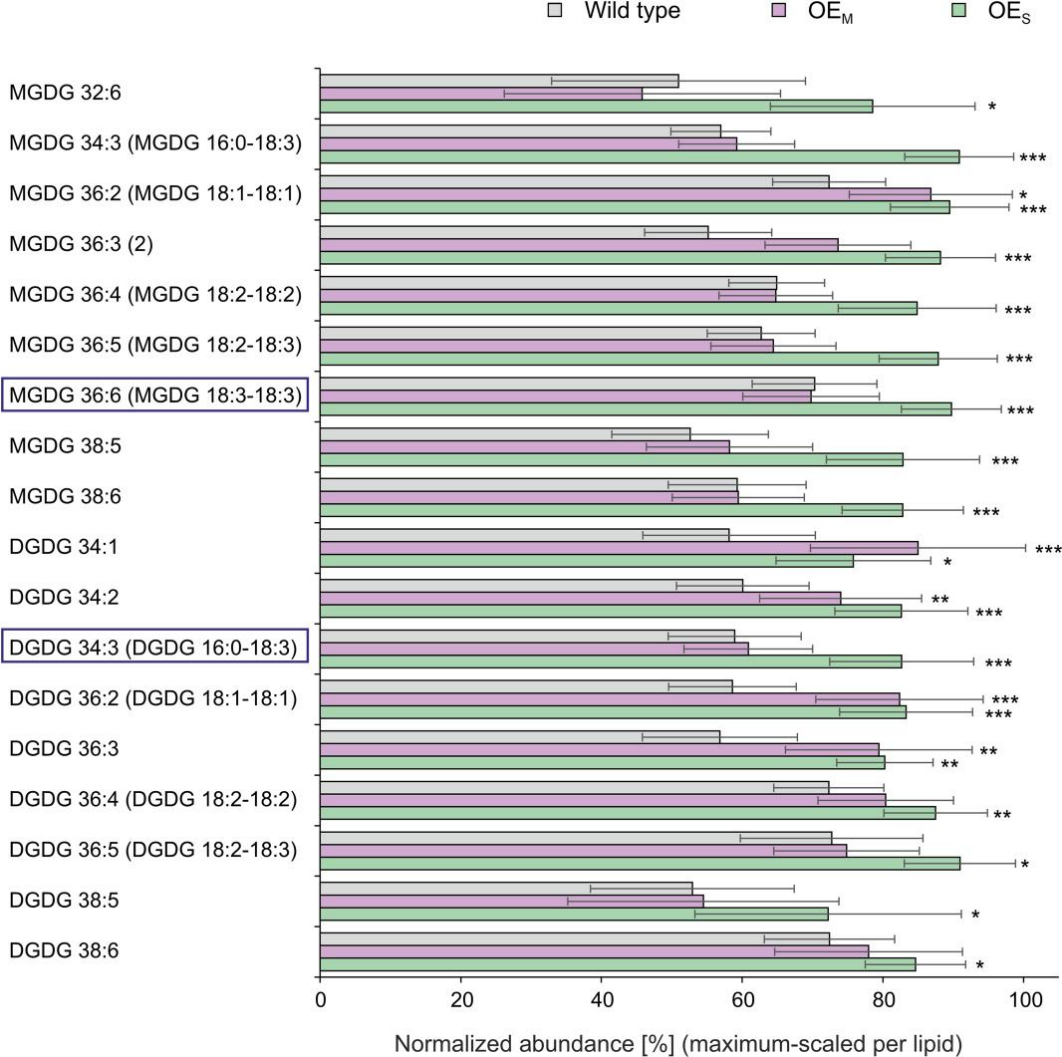
Accumulation of galactolipids in etiolated cotyledons of the wild type and the OE_S_ and OE_M_ transplastomic plants. Shown is a subset of MGDG and DGDG galactolipid species that show altered accumulation in etiolated OE_S_ cotyledons relative to the wild type. Lipid abundances were quantified by lipidomic profiling using LC-MS with normalization to sample fresh weight and the internal standard PC 34:0 (PC 17:0-17:0). Note that lipid abundances are maximum-scaled to allow for comparison of abundant and minor lipid species (and therefore, do not represent absolute abundances). Boxes indicate the most abundant galactolipids in etioplasts and during etioplast-to-chloroplast transition (Pipitone et al. 2021). Unless verified by co-chromatography of authenticated reference lipids, lipid species are named by lipid class, sum of carbon atoms in acyl chains, and number of unsaturations. Chromatographically separated structural isomers of lipids are indicated by numbers in parentheses. Data are means ± Sd (standard deviation) of 5 to 12 independent biological replicates each (see Materials and methods and Supplementary Data Set 1 for details). Asterisks indicate significant differences between the wild type and the OE_M_ or OE_S_ lines (Student's *t* test; *P < 0.05; **P < 0.01; ***P < 0.001). Note the enrichment of galactolipid species from the eukaryotic biosynthesis pathway. See text for details.

Many but not all species of MGDG and DGDG were significantly more abundant in etiolated OE_S_ seedlings than in the wild type. Some MGDGs and DGDGs were also increased in etiolated OE_M_ seedlings (Fig. 7; Supplementary Fig. S8 and Data Set 1). The increase of specific MGDG and DGDG species raised the question of a potential pathway preference for MGDG and DGDG synthesis in etiolated OE_S_ seedlings. We, therefore, co-analyzed synthetic MGDGs and defined biological galactolipids to annotate eukaryotic galactolipid species, such as 16:0-18:3, 18:1-18:1, 18:2-18:2, and 18:3-18:3, and prokaryotic galactolipids such as 16:3-18:2 and 16:3-18:3 from our lipidomic analyses (Armarego-Marriott et al. 2019), matching both the retention times and the exact molecular masses (Supplementary Data Set 1). While eukaryotic galactolipids were found to be overaccumulated (Fig. 7), prokaryotic galactolipids did not accumulate to higher levels in etiolated OE_S_ or OE_M_ seedlings (Supplementary Fig. S8).

**Figure 8.**
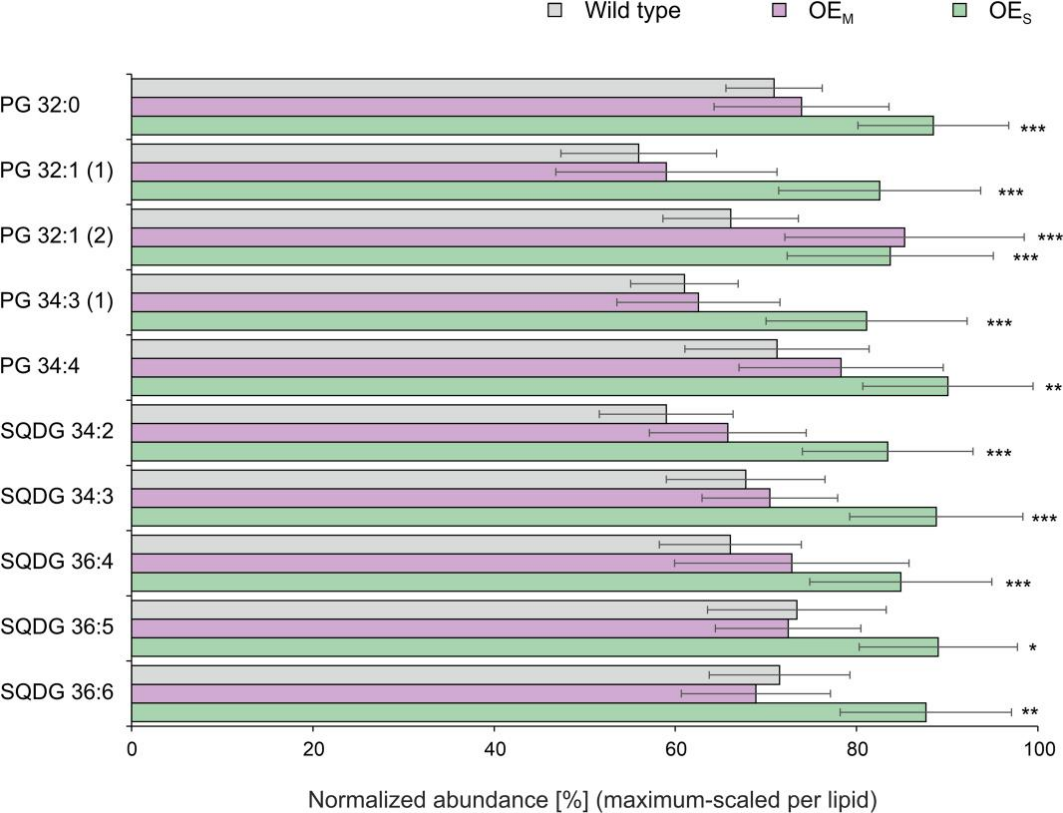
Accumulation of anionic plastid lipids in etiolated cotyledons of the wild type and the OE_S_ and OE_M_ transplastomic plants. Shown are the PG and SQDG lipid species that show altered accumulation in etiolated OE_S_ cotyledons relative to the wild type. Lipid abundances were quantified by lipidomic profiling using LC-MS with normalization to sample fresh weight and the internal standard PC 34:0 (PC 17:0-17:0). Lipid abundances are maximum-scaled to allow for comparison of abundant and minor lipid species. Lipid species are named by lipid class, sum of carbon atoms in acyl chains, and number of unsaturations. Chromatographically separated structural isomers of lipids are indicated by numbers in parentheses. Data are means ± Sd (standard deviation) of 5 to 12 independent biological replicates each (see Materials and methods and Supplementary Data Set 1 for details). Asterisks indicate significant differences between the wild type and the OE_M_ or OE_S_ lines (Student's *t* test; *P < 0.05; **P < 0.01; ***P < 0.001).

The 2 major classes of ionic lipids, PG and SQDG, each account for 5% to 10% of the total thylakoid lipids (Boudière et al. 2014). Unlike SQDG and galactolipids, PG is not specific to plastids, but is also synthesized in the ER and the mitochondrion (Hölzl and Dörmann 2019). Many but not all species of PG and SQDG were also found to overaccumulate in the etiolated OE_S_ seedlings relative to the wild type and some also in etiolated OEM seedlings (Fig. 8; Supplementary Fig. S9). Unavailability of PG and SQDG reference lipids did not allow unambiguous assignment of these lipid species to the prokaryotic or the eukaryotic pathway. The minor lipids PG 32:0 and PG 32:1 accumulated in the etiolated OES seedlings (Fig. 8). These PG species likely do not contain an 18-C fatty acid. Consequently, our data are also compatible with a contribution of the prokaryotic pathway to the stimulated lipid synthesis in etiolated OE_S_ seedlings, considering that PG is mostly synthesized by the prokaryotic pathway (Hölzl and Dörmann 2019).

Together, our data suggest that the content of (pro)thylakoid lipids is substantially elevated upon overexpression of PsbE in etioplasts. Increased thylakoid membrane protein synthesis in etioplasts appears to stimulate the production of thylakoid lipids, predominantly via the eukaryotic biosynthesis pathway, thus explaining the observed prothylakoid snailing phenotype in etioplasts of the OE_S_ seedlings. Our data also indicate that the abundance of membrane proteins in etioplasts limits prothylakoid biogenesis, in that prothylakoids are continuously made until all synthesized thylakoid proteins have been accommodated.

## Discussion

In this work, we have attempted to overexpress the enigmatic cytochrome *b*_559_ by manipulating the expression of the chloroplast operon that encodes its 2 apoproteins. Previous genetic manipulations of the 2 genes encoding the subunits of cytochrome *b*_559_ were focused on gene deletion from the plastid genome or site-directed mutagenesis of individual amino acid residues, especially the residues involved in heme coordination (Bock et al. 1994; Morais et al. 1998, 2001; Swiatek et al. 2003; Hamilton et al. 2014). Due to the difficulty to separate the essential role of cytochrome *b*559 in the early steps of PSII assembly from its possible physiological function in electron transfer, the insights gained from these approaches have remained limited. Our approaches to overexpress the apoproteins of cytochrome *b*559 resulted in a moderate increase in cytochrome *b*559 in both etioplasts and green tissues, but in a much more pronounced increase in PsbE protein accumulation in etioplasts. Our strong overexpression mutants also displayed pleiotropic phenotypes in the light, including pigment deficiency, PSII photoinhibition, and severe photooxidative damage that, at moderate to high light intensities, induced necrotic cell death (Figs. 2 and 6; Table 1; Supplementary Figs. S2, S3, and S7). To what extent these defects are caused by disturbed PSII assembly, impaired cytochrome *b*559 function and/or decreased cytochrome *b*_6_*f* complex contents (Hojka et al. 2014), currently cannot be resolved. The pleiotropic phenotypes (and the presence of a large fraction of free PsbE protein that is not assembled into functional cytochrome *b*559) currently preclude strong conclusions about cytochrome *b*559 function in the light.

The main objective of this work was to provide insights into the physiological relevance of cytochrome *b*_559_ accumulation in the dark. As etiolated seedlings are devoid of PSII (Armarego-Marriott et al. 2019; Fig. 4), it is unclear why cytochrome *b*559 accumulates in the dark. As the knockout of either of the 2 cytochrome *b*559 subunits leads to loss of autotrophic growth (Swiatek et al. 2003) and thus prevents seed production, seedling etiolation and deetiolation in the absence of the cytochrome currently cannot be studied. Therefore, overexpression appeared to be a promising strategy to investigate the functional consequences of altered cytochrome *b*559 accumulation in etioplasts. In this study, we have achieved a 1.44-fold increase of cytochrome *b*559 and an approximately 10-fold increase in PsbE protein levels in etioplasts of OE_S_ plants compared to the wild type. While the cytochrome *b*559 accumulating in the dark could solely serve as nucleus for later PSII assembly upon illumination, it seems also possible that it serves a specific physiological function and/or structural role in etioplasts. For example, cytochrome *b*559 could participate in redox control of the PQ pool in etioplasts via the oxidation of plastoquinol. However, another plastoquinol oxidase (PTOX, the plastid terminal oxidase, a homolog of mitochondrial alternative oxidases) accumulates in etioplasts (Lennon et al. 2003). PTOX was proposed to participate in redox control in etioplasts by delivering electrons from the PQ pool to oxygen (Kambakam et al. 2016). In order to test whether PTOX accumulation is affected by the overaccumulation of cytochrome *b*559, we determined PTOX protein amounts in etiolated wild type and OES seedlings (Supplementary Fig. 10A). Unaltered PTOX levels suggest that PTOX accumulation is largely independent of cytochrome *b*559.

A striking finding of our present study was that cytochrome *b*_559_ levels were only moderately increased in etiolated OE_S_ seedlings, while the PsbE apoprotein overaccumulated to 10-fold higher levels than in the wild type. Recently, the protein RESISTANCE TO PHYTOPHTHORA1 (RPH1) was reported to facilitate cytochrome *b*_559_ assembly during early PSII biogenesis, possibly by mediating heme insertion into the PsbE-PsbF apoprotein (Che et al. 2024). Whether or not RPH1 (or other assembly factors) limits the accumulation of cytochrome *b*_559_ in our OE_S_ transplastomic tobacco lines remains to be investigated.

**Figure 9.**
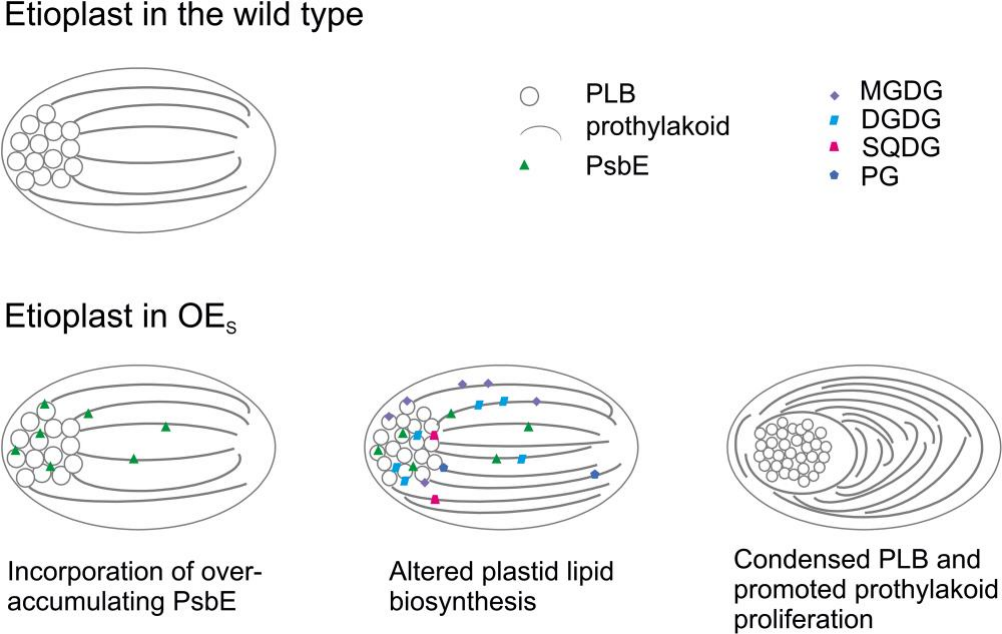
Overexpression of PsbE promotes prothylakoid formation in etioplasts by altering plastid lipid biosynthesis. Model illustrating how the incorporation of overaccumulating PsbE leads to a condensed PLB structure, stimulated plastid lipid biosynthesis, and prothylakoid proliferation. For simplicity, only supernumerary PsbE and lipid molecules are indicated. See text for details.

Our findings suggest 2 important structural functions of PsbE and/or cytochrome *b*_559_ in etioplasts: (i) a contribution to the paracrystalline order of the PLB structure (Fig. 5, B and C) and (ii) a rate-limiting role in prothylakoid biogenesis (Figs. 5, A and B, and 9). The strong overexpressors (OE_S_ plants) show a massive proliferation of prothylakoid membranes in etioplasts, causing a striking membrane snailing phenotype (Fig. 5). The very strong overaccumulation of PsbE (in contrast to the moderate overaccumulation of the fully assembled cytochrome *b*_559_) leads us to propose that the PsbE apoprotein is more likely to be causally responsible for the membrane snailing phenotype. Nevertheless, taking into account the strong transcriptional overaccumulation of *psbF*-*L*-*J* (that are part of the same operon), we cannot fully rule out the possibility that overexpression of PsbF, PsbL, and/or PsbJ also contributes to the observed ultrastructural phenotype. Together with our analysis of lipid accumulation in etioplasts (Supplementary Data Set 1), these observations suggest that membrane protein provision represents a rate-limiting step in the biogenesis of prothylakoids in etioplasts (Fig. 9). The prothylakoid snailing observed in etioplasts of our transplastomic OE_S_ seedlings is somewhat reminiscent of the phenotype seen upon overexpression of Tic40, a component of the protein import machinery in the inner envelope membrane of the chloroplast. Tic40 overaccumulation in tobacco chloroplasts led to proliferation of the inner membrane and formation of a multilayer envelope structure (Singh et al. 2008). Thus, regulation of membrane biogenesis by protein provision could be a more general theme in the biosynthesis of biological membrane systems. It would be interesting to overexpress other thylakoid membrane proteins in etioplasts and analyze the consequences at the ultrastructural level. However, our efforts to achieve overexpression of other thylakoid proteins have been unsuccessful. This is unsurprising, given that cytochrome *b*559 is the only small protein subcomplex known to accumulate in etioplasts. Although the NADH dehydrogenase complex, the ATP synthase, and the cytochrome *b*_6_*f* complex also accumulate in the dark, the individual subunits of these large complexes are not stable unless faithfully assembled into the complexes (e.g. Shikanai et al. 1998; Hager et al. 1999). Thus, overexpression of most, if not all, subunits of one of these complexes is likely required to obtain another case of prothylakoid membrane snailing.

Our lipid profiling experiments revealed that PsbE overaccumulation leads to a strong stimulation of plastid lipid synthesis (Figs. 7 and 8). In plants, de novo fatty acid biosynthesis occurs in plastids, where the stromal enzyme acetyl-CoA carboxylase (ACCase) catalyzes the carboxylation of acetyl-CoA to produce malonyl-CoA. The ACCase is a highly regulated enzyme (Salie and Thelen 2016; Caroca et al. 2021) that is believed to be largely inactive in the dark, due to redox regulation mediated by light and the thioredoxin system (Sasaki et al. 1997). Fatty acid elongation in the plastid occurs up to a chain length of 16 or 18 carbon atoms, and while part of the C16 and C18 fatty acids are directly utilized by 16:3 plants in the plastid for lipid synthesis (by the so-called prokaryotic pathway), a large fraction becomes exported to the ER for further chain elongation, desaturation, and lipid biosynthesis via the so-called eukaryotic pathway. Part of the lipid backbones assembled by the eukaryotic pathway are then reimported into plastids and used for the biosynthesis of plastid lipids, including the 2 major lipid classes of the thylakoid membrane, the galactolipids MGDG and DGDG (Ohlrogge and Browse 1995; Li et al. 2016; Hölzl and Dörmann 2019). Our lipidomic analysis revealed a particularly pronounced overaccumulation of galactolipids of the eukaryotic pathway (Fig. 7) that contain C18 fatty acids at the sn-2 position, which was further confirmed by DAG species annotation using synthetic MGDG 18:1-18:1, MGDG 18:2-18:2, and MGDG 18:3-18:3.

Enhanced lipid accumulation in etiolated OE_S_ seedlings was different from transgenic enhancement of the eukaryotic lipid biosynthesis pathway in tobacco chloroplasts (Fritz et al. 2007). The latter resulted in increased PC and reduced MGDG and DGDG contents in green leaves and caused growth defects and reduction of the chlorophyll content per leaf area. Thylakoids of these mutants accumulated DGDG and PG, but showed decreased MGDG and SQDG, with accumulation of 36:6 at the expense of 34:6 and 34:3 DAG species of both galactolipids (Fritz et al. 2007). In this context, it is important to note that chloroplasts and etioplasts differ substantially in membrane structures and the mode of carbon supply for lipid biosynthesis. Given that the plastid-localized fatty acid biosynthesis is downregulated in the dark by redox-mediated inactivation of ACCase (Sasaki et al. 1997), we propose that prokaryotic lipid biosynthesis in etiolated OES seedlings is limited, and therefore, the eukaryotic pathway is preferentially utilized to support lipid provision for prothylakoid snailing.

How the abundance of thylakoid proteins regulates lipid biosynthesis in etioplasts is an interesting question that warrants further investigation. In the simplest scenario, the synthesized extra membrane proteins represent a sink for thylakoid lipids (Fig. 9), thus limiting lipid availability to other cellular processes such as membrane biogenesis and/or the biosynthesis of lipid-derived plant hormones (e.g. jasmonates). These deficits could then be sensed and translated into a stimulation of fatty acid and lipid biosynthetic pathways at the level of gene expression and/or the level of enzyme activity. To gain preliminary insights in the relationship between lipid and protein accumulation, we determined the total lipid to total protein ratios (Supplementary Fig. 10B). The ratios were not significantly different between etiolated seedlings of the wild type and the OE_S_ mutants, providing further circumstantial evidence for a coordinated regulation of protein and lipid contents.

Thylakoid membranes are a highly protein-rich membrane system, the formation of which requires the concerted synthesis and assembly of membrane proteins and lipids. The formation of prothylakoids in the dark is likely useful to accelerate the assembly of the photosynthetic apparatus upon illumination and accommodate the ATP synthase and other membrane protein complexes that accumulate in the dark (Floris and Kühlbrandt 2021). Cytochrome *b*559 seems to be a particularly suitable component to meet the requirement for membrane protein provision for prothylakoid biogenesis. This is because (i) unlike many other thylakoid proteins, cytochrome *b*559 does not bind pigments and, therefore, its accumulation does not entail the risk of harmful photochemistry upon light exposure, and (ii) the cytochrome is required early in PSII assembly (Müller and Eichacker 1999; Chu and Chiu 2016), and thus, its presence already in the dark can speed up photosystem biogenesis during deetiolation. It seems possible that the coordination of membrane protein synthesis with membrane lipid biosynthesis also applies to thylakoid biogenesis in chloroplasts, in that the total size of the thylakoid network is also controlled by membrane protein provision. However, due to the instability of individual (unassembled) subunits of the thylakoid protein complexes, and the difficulties associated with stable overexpression of entire complexes, this hypothesis currently cannot be easily tested.

In summary, our work described here (i) provides evidence for a structural role of PsbE and possibly cytochrome *b*_559_ in PLB formation and prothylakoid biogenesis and (ii) demonstrates the potential for lipid synthesis to be controlled by the amount of membrane proteins provided.

## Materials and methods

### Plant material and growth conditions

Tobacco (*N. tabacum* cv. Petit Havana) seeds used in this study were harvested from plants grown under standard greenhouse condition (16 h light at 25 °C, 8 h dark at 20 °C; average light intensity: 270 *µ*mol photons m^−2^ s^−1^). For germination under aseptic conditions, seeds were surface-sterilized with hypochlorite solution and sown on Murashige and Skoog (MS) medium (Duchefa Biochemie) supplemented with 1% (w/v) sucrose. Tobacco plants used for phenotypic analyses were initially grown under nursery conditions (16 h light at 22 °C and 8 h dark at 18 °C; light intensity: 200 *µ*mol photons m^−2^ s^−1^) and subsequently transferred to standard growth condition (16 h light at 25 °C and 8 h dark at 20 °C) with the light intensities indicated. For measurements of photosynthetic parameters, plants were transferred from the nursery conditions to a controlled environment chamber (Conviron) with 300 *µ*mol photon light intensity, 22 °C temperature and 75% relative humidity during the 16 h day, and 18 °C and 70% relative humidity during the night. For (de)etiolation experiments, the sucrose was removed from the synthetic medium. Etiolation (7 to 8 days in darkness at 25 °C) was preceded by short illumination of the imbibed seeds (120 *µ*mol photons m^−2^ s^−1^ at 25 °C) for 45 min to induce germination. Etiolated samples were harvested in darkness or under dim green light. For lipidomic analyses, tobacco seedlings were grown vertically under aseptic conditions, the cotyledons with ∼2 mm hypocotyl from etiolated seedlings were pooled, and each pool represented a biological replicate. For the wild type, 5 biological replicates were analyzed, and for the OE_S_ and OE_M_ transplastomic lines, 6 biological replicates were analyzed from 2 independent transplastomic lines each (OE_M_-1 and OE_M_-2 and OE_S_-1 and OE_S_-2).

### Construction of plastid transformation vectors for cytochrome *b*_559_ overexpression

The plastid transformation vectors for overexpression of cytochrome *b*_559_ (Fig. 1A) were constructed as follows. A 2.2 kb region of the tobacco plastid genome (ptDNA) containing the *psbEFLJ* operon and the entire reading frame of the upstream gene *petL* was excised by digestion with the restriction enzymes MluI and ApaI, followed by a fill-in reaction using the Klenow fragment of *Escherichia coli* DNA polymerase I to generate blunt ends. The fragment was then inserted into the cloning vector pBlueScript II SK(+) that had been linearized with the restriction enzyme Ecl136II. The resultant vector was linearized with the restriction enzyme NsiI, and blunt ends were produced by treatment with T4 DNA polymerase. Subsequently, a PCR-amplified *aadA* cassette driven by the promoter and 5′ UTR from the chloroplast *psbA* gene and the 3′UTR from the chloroplast *rbcL* gene of *Chlamydomonas reinhardtii* (Fleischmann et al. 2011) was introduced by ligation, creating vector pTAM5 (Fig. 1A). Vector pTAM2 was created by the same process, but with a PCR product containing an additional tobacco *Prrn* promoter directly 3′ to the resistance cassette. To generate vector pTAM6, plasmid pTAM5 was linearized with Ecl136II, and a PCR-amplified sequence containing the *Prrn* promoter coupled to the leader sequence from the bacteriophage T7 *gene10* (*T7g10*) was inserted. All 3 vectors contain more than 400 bp of unmodified ptDNA at the left and right borders to aid integration into the plastid genome by homologous recombination. The sequences of all final plastid transformation vectors were confirmed by complete resequencing. Oligonucleotides used as amplification primers are listed in Supplementary Table S1.

### Plastid transformation and selection of homoplasmic transplastomic lines

Tobacco leaves from aseptically grown *N. tabacum* cv. Petit Havana plants were harvested and bombarded with vector DNA-coated 0.6 *µ*m gold particles using the PDS-1,000/He biolistic gun (Bio-Rad). Bombarded leaves were then cut into pieces of approximately 5 × 5 mm and exposed to selection on an MS-based plant regeneration medium containing 500 mg L^−1^ spectinomycin (Svab and Maliga 1993). Primary spectinomycin-resistant shoots were subjected to an additional round of regeneration on plant regeneration medium supplemented with ^50^0 mg L^−1^ spectinomycin to select for homoplasmic lines. Regenerated homoplasmic shoots were rooted on hormone-free MS medium supplemented with ^50^0 mg L^−1^ spectinomycin, transferred to soil, and grown to maturity for seed production under standard greenhouse conditions.

### Inheritance assays

To test for maternal transgene inheritance and confirm homoplasmy of the transplastomic OE_W_, OE_M_, and OE_S_ lines, T1 seeds were surface-sterilized by soaking in hypochlorite solution and sown on agar-solidified MS medium containing 500 mg L^−1^ spectinomycin. Homoplasmy was evidenced by growth of a homogenous population of antibiotic-resistant seedlings (Fig. 1C; Supplementary Fig. S1).

### Nucleic acid isolation and hybridization procedures

Leaf material or whole seedlings were snap-frozen in liquid nitrogen and used for extraction of nucleic acids. For total cellular DNA isolation, a cetyltrimethylammonium bromide–based method was used (Doyle and Doyle 1990). Total RNA was extracted using the NucleoSpin RNA Plant kit, according to the manufacturer's instructions (Macherey-Nagel). For Restriction Fragment Length Polymorphism (RFLP) analysis, 1.5–4.0 *μ*g total DNA was digested with the restriction enzyme BsaI, separated by electrophoreses in 1% (w/v) agarose gels, and transferred onto Hybond-XL nylon membranes (GE Healthcare) by capillary blotting. For RNA gel blot analyses, samples of total cellular RNA were electrophoretically separated in 1% (w/v) denaturing agarose gels and blotted onto Hybond-XL nylon membranes (GE Healthcare). Nucleic acids were covalently cross-linked to the dried membrane by UV light. Gel-purified PCR products amplified from total tobacco DNA were used as probes for RFLP and RNA gel blot analyses. The fragments were radiolabeled with [α-32P] dCTP by random priming (GE Healthcare). Hybridizations were performed at 65 °C.

### Thylakoid membrane isolation

For preparation of thylakoid membranes, leaf tissue was harvested and ground in liquid nitrogen in the dark or under dim light in homogenization buffer (50 mm HEPES pH 7.5, 330 mm sorbitol, 1 mm MgCl_2_, 2 mm EDTA, 5 mm sodium ascorbate, 0.1% [w/v] BSA). The homogenate was filtered through 2 layers of Miracloth (Merck) and centrifuged at 5,000 × *g* for 10 min at 4 °C in the dark. The pellet was then gently resuspended in shock buffer (50 mm HEPES pH 7.5, 5 mm sorbitol, 5 mm MgCl_2_), the samples were centrifuged at 5,000 × *g* for 10 min at 4 °C, and the pellet obtained was washed and resuspended in storage buffer (50 mm HEPES pH 7.5, 100 mm sorbitol, 10 mm MgCl_2_).

### Protein extraction and immunoblot analyses

Total protein extracts were prepared from approximately 100 mg of leaf tissue. The tissue was ground to a fine powder in liquid nitrogen and resuspended in protein extraction buffer (20 mm HEPES [pH 7.5], 2 mm EDTA [pH 7.4], 2 mm EGTA [pH 7.4], 20 mm NaF, 1 mm Na_3_VO_4_, 10% [v/v] glycerol, 100 mm NaCl, 0.2% [v/v] Triton X-100, and 1× complete protease inhibitor cocktail [Roche]) at a tissue-to-buffer ratio of 1:3. The supernatant was collected after 2 centrifugations at 13,000 rpm for 10 min each in a tabletop centrifuge. Protein concentrations were determined using the Pierce BCA Protein Assay Kit (Thermo Fisher Scientific). The desired amount of protein was mixed with 4× SDS sample buffer (200 mm Tris-HCl, pH 6.8, 400 mm DTT, 8% [w/v] SDS, 0.4% [w/v] bromophenol blue, 40% [v/v] glycerol) and denatured for 10 min at 95 °C. Equal amounts of proteins were separated by 12% or 15% SDS-polyacrylamide gel electrophoresis (SDS-PAGE) and blotted onto polyvinylidene fluoride membranes (GE Healthcare). All antibodies used in this study are listed in Supplementary Table S2. For relative quantitation of PsbE contents, band intensities of the dilution series were determined using the ImageJ software.

### Deetiolation experiments and determination of greening rates

To determine the greening rates of etiolated tobacco seedlings, more than 100 seeds per sample (wild type and cytochrome *b*_559_ overexpression lines) were germinated and grown in darkness for 7 to 8 days (see above). For deetiolation, the seedlings were transferred to continuous white light (120 *µ*mol photons m^−2^ s^−1^) for different time periods. The greening rate was calculated as the chlorophyll content of deetiolating seedlings divided by the chlorophyll content of seedlings grown in the light.

### Complementary DNA (cDNA) synthesis and RT-qPCR

One microgram of extracted total RNA was reverse transcribed using the SuperScript III Reverse Transcriptase kit (Thermo Fisher Scientific) and (dT)_20_ as primer. RT-qPCR was performed using LightCycler SYBR Green reaction mixtures (Roche Applied Science) on a LightCycler 480 Real-Time PCR System. Relative transcript levels were calculated using the comparative CT method (ΔΔCT) and normalized to the transcript levels of *ACTIN* (U60489). The primers used in this study are listed in Supplementary Table S1.

### Microscopic analyses and quantification of plastid ultrastructure

TEM analysis was done as previously described for seedlings grown on sucrose-depleted synthetic medium (Bykowski et al. 2020). Cotyledons with up to 2 mm hypocotyl attached were fixed for 2 h in 2.5% (w/v) glutaraldehyde in 50 mm cacodylate buffer (pH 7.4), postfixed in 2% (w/v) osmium tetroxide at 4 °C overnight, and dehydrated in a graded series of acetone using the following sequence: 10 min, 30% (v/v); 10 min, 40% (v/v); 10 min, 60% (v/v); 30 min, 70% (v/v); 40 min, 80% (v/v); and 3 × 40 min, 100%. Dehydrated samples were infiltrated in acetone:resin mixtures (3:1; 1:1; 1:3) and finally embedded in a pure resin medium (Agar 100 Resin Kit). Blocks with samples were cut into 90 nm specimens using a Leica UCT ultramicrotome. TEM images were produced with the JEM 1400 (JEOL) instrument equipped with a Morada G2 CCD camera (EMSIS GmbH) in the Laboratory of Electron Microscopy, Nencki Institute of Experimental Biology of the Polish Academy of Sciences, Warsaw, Poland.

The ultrastructural characteristics of PLBs were analyzed with the help of the ImageJ software. The periodicity parameter was calculated based on averaged values obtained from Fast Fourier Transform of 2D cross-sections of micrographs showing nearly hexagonal planar lattices. The length of the prothylakoids (PTs) was manually measured on micrographs using the segmented line function of the ImageJ software, and the number of PT-PLB connections was counted directly from the images of whole etioplasts.

### Lipidomic analysis

Changes of lipid abundances were quantified by lipidomic profiling using liquid chromatography-tandem mass spectrometry (LC-MS) as initially described by Giavalisco et al. (2011) and subsequently modified for *tobacco (N. tabacum* cv. “Petit Havana”) leaf samples (Armarego-Marriott et al. 2019). Briefly, samples of ∼30 ± 5 mg (fresh weight) of snap-frozen, pooled, and homogenized tissue from harvested cotyledons with up to 2 mm attached hypocotyl material were extracted by the methanol:methyl-tert.-butyl-ether(MTBE):water protocol, as described previously (Giavalisco et al. 2011). Lipids were extracted with 1 mL MTBE:methanol (3:1, v/v) that contained 0.5 *µ*g mL^−1^ 1,2-diheptadecanoyl-sn-glycero-3-phosphocholine, PC 17:0-17:0 (Sigma-Aldrich/Avanti Polar Lipids, 850360P), as internal standard. Samples were rapidly mixed with the solvent and incubated for 10 min in an orbital shaker followed by 10 min sonication at ≤4 °C. Phase separation was induced by mixing with 0.5 mL water:methanol (3:1, v/v, UPLC MS grade; BioSolve, Dieuze, France) and 5 min centrifugation at 16,000 × *g* at 4 °C. The nonpolar, upper phase (∼500 mL) was vacuum concentrated and stored at −80 °C until use. Dried lipid samples were redissolved in ∼500 *µ*L buffer B (see below).

Ultra performance liquid chromatography (UPLC) separation of 2 *µ*L redissolved lipid samples was performed by an Acquity UPLC system with an BEH C_8_ reversed-phase column (100 mm × 2.1 mm with 1.7 *µ*m particles; Waters GmbH, Eschborn, Germany, http://www.waters.com) with temperature control set to 60 °C. The mobile phases were as follows: buffer A: water (UPLC MS grade; BioSolve) with 1% (v) 1 m NH_4_Ac, 0.1% (v) acetic acid and buffer B: acetonitrile:isopropanol (7:3, v/v, UPLC grade; BioSolve) containing 1% (v) 1 m NH_4_Ac and 0.1% (v) acetic acid. The UPLC was operated at 400 *μ*L min^−1^ flow rate with 1 min 45% (v) buffer A, a 3 min linear gradient from 45% to 25% buffer A, an 8 min linear gradient from 25% to 11% buffer A, a 3 min linear gradient from 11% to 1% buffer A, and a final 3 min wash with 0% buffer A (i.e. 100% buffer B). After resetting to 45% buffer A, the column was equilibrated for 4 min, amounting to 22 min total run time (Pant et al. 2015).

Mass spectrometric analysis of 1 to 17 min of the UPLC gradient was performed with an Exactive mass spectrometer (Thermo Fisher, Waltham, USA, http://www.thermofisher.com) with m/z range 150 to 1500 at 10,000 resolution and 10 scans s^−1^. The loading time of the mass spectrometer was set to 100 ms. The capillary voltage was 3 kV with a sheath gas flow of 60 and an auxiliary gas flow of 35 (arbitrary units). The capillary temperature was set to 150 °C, with drying gas in the heated electrospray source at 350 °C. The skimmer and tube lens voltages were 25 and 130 V, respectively. Each sample was measured twice, once in positive and once in negative-ionization mode.

Data processing of the chromatogram raw files with baseline correction, chemical noise subtraction, alignment, and peak detection was done with the GeneData REFINER MS 5.3 software (https://www.genedata.com/), as previously described (Giavalisco et al. 2011). Mass features characterized by 2 orthogonal characteristics, namely, mass-to-charge ratio (m/z) and retention time (RT), were assembled into a numerical data matrix with respective abundances (arbitrary units) from each sample. Lipids were annotated by matching to a reference library of expected m/z and RT values (Armarego-Marriott et al. 2019) and co-chromatography of MGDG and DGDG reference samples (see below). Quantified monoisotopic mass features, i.e. lip_i_d adduct ions [M + NH4]+ (positive ioniz^a^tion mode) or [M-H]− (negative ionization mode), with match qualities of m/z and RT are reported in Supplementary Data Set 1. This lipidomic method distinguishes lipid classes, the sum of carbon atoms and the degree of unsaturation in DAG moieties. We name lipids accordingly, e.g. PC 34:0 for the internal standard 1,2-diheptadecanoyl-sn-glycero-3-phosphocholine. Lipid isomers can be chromatographically resolved. In such cases, we use name extensions, i.e. (1), (2), etc., in chromatographic order. To characterize the acyl-chain composition of monitored lipids, we performed co-chromatography with commercially available authenticated MGDG and DGDG preparations, namely, synthetic MGDG 18:1-18:1 (Sigma-Aldrich/Avanti Polar Lipids, 840531P), MGDG 18:2-18:2 (Sigma-Aldrich/Avanti Polar Lipids, 840532P), and MGDG 18:3-18:3 (Sigma-Aldrich/Avanti Polar Lipids, 840533P), and defined biological mixtures of MGDGs (Sigma-Aldrich/Avanti Polar Lipids, 840523P) and DGDGs (Sigma-Aldrich/Avanti Polar Lipids, 840524P), according to the manufacturer’s analysis certificates. In these cases, we use additional name extensions with acyl chain designations, e.g. PC 34:0 (PC 17:0-17:0) or MGDG 36:6 (MGDG 18:3-18:3).

Relative lipid abundances were quantified after background subtraction of initial mass feature abundances (arbitrary units) using nonsample controls, normalization to the abundance of the internal standard PC 34:0 (PC 17:0-17:0) and to sample fresh weight (g) of each sample. For the comparison of abundant and minor lipid species, we used maximum scaling of each lipid (i.e. % abundances of each sample relative to the maximum abundance of each lipid species across all samples). Statistical testing of 5 to 12 independent biological replicates per genotype and condition was performed by 2-tailed, heteroscedastic Student’s *t* tests with the significance thresholds *P* < 0.05, *P* < 0.01, and *P* < 0.001.

### Spectroscopic methods

Leaf chlorophyll contents and chlorophyll *a*/*b* ratios were determined from leaf extracts in 80% (v/v) acetone (Porra et al. 1989) with a V-730 UV-Vis Spectrophotometer (Jasco GmbH). Chlorophyll-*a* fluorescence of intact leaves was measured at 22 °C using a Dual-PAM-100 instrument (Heinz Walz GmbH). After 30 min of dark adaptation, F_V_/F_M_ and light-response curves of the chl*o*rophyll-*a* fluorescence parameters NPQ, qL, and Y(NO) were determined. Under light-limited conditions below the growth light intensity, actinic light intensity was increased in 150 s intervals, while under light-saturated conditions, the light intensity was increased every 60 s.

Contents of PSII, cytochrome *b*_6_*f* complex, and PSI were quantified in thylakoids isolated as described previously (Schöttler et al. 2004). PSII and cytochrome *b*_6_*f* complex contents were determined from difference absorbance spectra of cytochromes *f* and *b*_6_ (cytochrome *b*_6_*f* complex) and the high- and low-potential forms of cytochrome b559 (PSII complex) using a V-550 spectrophotometer (Jasco) equipped with a head-on photomultiplier, as previously described (Kirchhoff et al. 2002; Hojka et al. 2014). PSI quantification in isolated thylakoids was done with the plastocyanin-P700 version of the Dual PAM (Heinz Walz GmbH) using light-induced difference absorbance signals of P700 in the presence of 10 mm sodium ascorbate as electron donor and 100 *µ*M methylviologen as electron acceptor (Schöttler et al. 2007). A F-6500 fluorometer (Jasco Inc., Groß-Umstadt, Germany) was used to measure 77 K chlorophyll-a fluorescence emission spectra. Freshly isolated thylakoid membranes equivalent to 10 *μ*g chlorophyll mL^−1^ were excited at 430 nm wavelength with a bandwidth of 10 nm, and the emission spectrum was recorded between 655 and 800 nm wavelength in 0.5 nm intervals with a bandwidth of 1 nm.

### Etioplast isolation and quantification

To obtain a sufficiently large amount of etiolated material for etioplast isolation, nearly mature tobacco plants grown under long-day conditions were kept in darkness for 5 days (wild type) or 7 days (OE_S_). During that period, cell division and expansion of the smallest, developing leaves results in the growth of new, pale tissue, as previously described (Armarego-Marriott et al. 2019). Etioplasts were isolated based on previously reported methods (Eichacker et al. 1996) with minor modifications. Briefly, >60 g of new leaves that had emerged in the dark (Armarego-Marriott et al. 2019) was collected from the wild type and the strong cytochrome *b*_559_ overexpression plants (OE_S_-1 and OE_S_-2) under dim light, homogenized in precooled buffer A (0.4 m sorbitol, 2 mm EDTA, 50 mm HEPES, pH 8.0) in a Waring blender. The homogenate containing the released intact etioplasts was then filtered through 20 *µ*m nylon net filters (Merck Millipore Ltd.) and centrifuged (4,097 × *g*, 3 min). The pellets were resuspended in 5 mL of buffer A (per 60 g of starting material), and intact etioplasts were purified by centrifugation in a 35%/65% (v/v) Percoll step gradient. After collection of the intact etioplasts from the interface, the samples were diluted and washed gently in 45 mL of Buffer B (0.4 m Sorbitol, 50 mm HEPES, pH 8.0) and centrifuged at 4,097 × *g* for 2 min. The washed plastids were resuspended thoroughly using cut pipet tips in an appropriate amount of buffer B. Samples (5 *µ*L) from each isolation were taken for quantification using a phase contrast microscope (Epi-Fluorescence BX-51, Keyence Deutschland GmbH). For quantification of the number of plastids in each isolation, at least 10 images were analyzed.

### Total lipid isolation and quantification

Total lipids were isolated according to published protocols (Shiva et al. 2018). Briefly, samples of 30 mg etiolated leaf material were homogenized in a CryoMill (Retsch GmbH). The powder was suspended in 400 *µ*L of precooled isopropanol supplemented with 0.01% (v/v) butylated hydroxytoluene and incubated in a shaker at 1,400 rpm at 75 °C for 15 min. After cooling to room temperature, 1,200 *µ*L of chloroform:methanol:water mixture (30/41.5/3.5, v/v/v) was added, and samples were incubated for 24 h at 300 rpm at 25 °C. Lipids were collected by centrifugation at 13,000 rpm for 15 min at room temperature. The resultant supernatant was transferred into a clean 2 mL Eppendorf tube, and lipids were then concentrated in a SpeedVac. The amount of total lipids was quantified using the LipoQ Lipid Quantification Assay (HiSS Diagnostics) according to the manufacturer's instructions.

### Accession numbers

Sequence data from this article can be found in the GenBank data libraries under following accession numbers: PsbE (800531/NP_054517.1), PsbF (800531/NP_054516.1), PsbL (800491/NP_054515.2), PsbJ (800496/NP_054514.1), PetL (800424/NP_054519.1), ZAT12 (XM_016627562.1), WRKY40 (XM_016601521.1), APXc (AF443182), CAT1 (U93244), and BAP1 (XM_016622977.1). Other relevant sequence data can be obtained via GenBank under the accession number NC_001879.2.

## Supplementary Material

koae259_Supplementary_Data

## Data Availability

The data underlying this article are available in the article and in its online supplementary material.

## Supplementary data

The following materials are available in the online version of this article.

**Supplementary Figure S1.** Inheritance assays demonstrating homoplasmy of an additional set of transplastomic lines for the 3 overexpression constructs.

**Supplementary Figure S2.** Comparison of OE_S_ plants grown under 25 *µ*mol photons m^−2^ s^−1^ or 100 *µ*mol photons m^−2^ s^−1^ light intensity.

**Supplementary Figure S3.** Photosynthetic parameters under different light intensities in wild type and cytochrome *b*5_59_ overexpression plants.

**Supplementary Figure S4.** 77 K chlorophyll-*a* fluorescence emission spectra of the wild type, 2 independent OE_W_ and OE_M_ lines each, and 3 independent OE_S_ lines.

**Supplementary Figure S5.** Analysis of *petA* mRNA accumulation in OE_W_, OE_M_, and OE_S_ transplastomic plants in comparison to wild-type control plants.

**Supplementary Figure S6.** Plastid ultrastructure in cotyledons of 7-day-old etiolated wild type and OE_S_ seedlings.

**Supplementary Figure S7.** Increase in chlorophyll content in the wild type and the transplastomic OE_W_, OE_M_, and OE_S_ plants during deetiolation.

**Supplementary Figure S8.** Galactolipids with largely unaltered accumulation in etiolated cotyledons of the OE_S_ and OE_M_ transplastomic plants relative to the wild type.

**Supplementary Figure S9.** Anionic plastid lipids (PG and SQDG) with largely unaltered accumulation in etiolated cotyledons of the OE_S_ and OE_M_ transplastomic plants relative to the wild type.

**Supplementary Figure S10.** Determination of PTOX protein amounts and total lipid/total protein ratios in wild type and OE_S_ plants.

**Supplementary Table S1.** List of oligonucleotides used as PCR primers in this study.

**Supplementary Table S2.** List of antibodies used in this study.

**Supplementary Data Set 1.** Lipidomic analysis results of plastid lipids and lipid classes with quality parameters of lipid annotations.

## Dive Curated Terms

The following phenotypic, genotypic, and functional terms are of significance to the work described in this paper:

HEPES CHEBI: CHEBI:46756
