# Supplementary material for: Membrane protein provision controls prothylakoid biogenesis in tobacco etioplasts
Source: Plant Cell. 2024 Sep 25;36(12):4862–80. doi: 10.1093/plcell/koae259 (PMC11638105; doi:10.1093/plcell/koae259)
Supplement: koae259_Supplementary_Data [file koae259_supplementary_data.zip › Bock_Suppl Mat_revised_clean_BL_RB.pdf]

SUPPLEMENTARY MATERIALS

SUPPLEMENTARY FIGURES

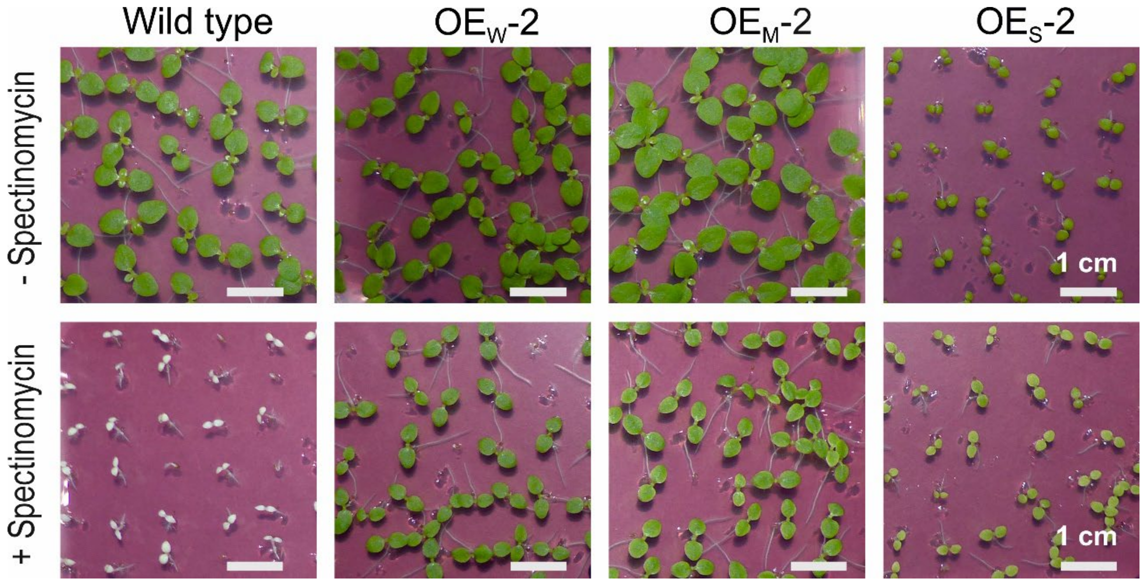

Supplementary Figure S1. Inheritance assays demonstrating homoplasmy of an additional set of transplastomic lines for the three overexpression constructs. (Supports **Figure 1**)
OE$_W$: weak overexpressing line, OE$_M$: medium overexpressing line, OE$_S$: strong overexpressing line. For details, see Figure 1C.

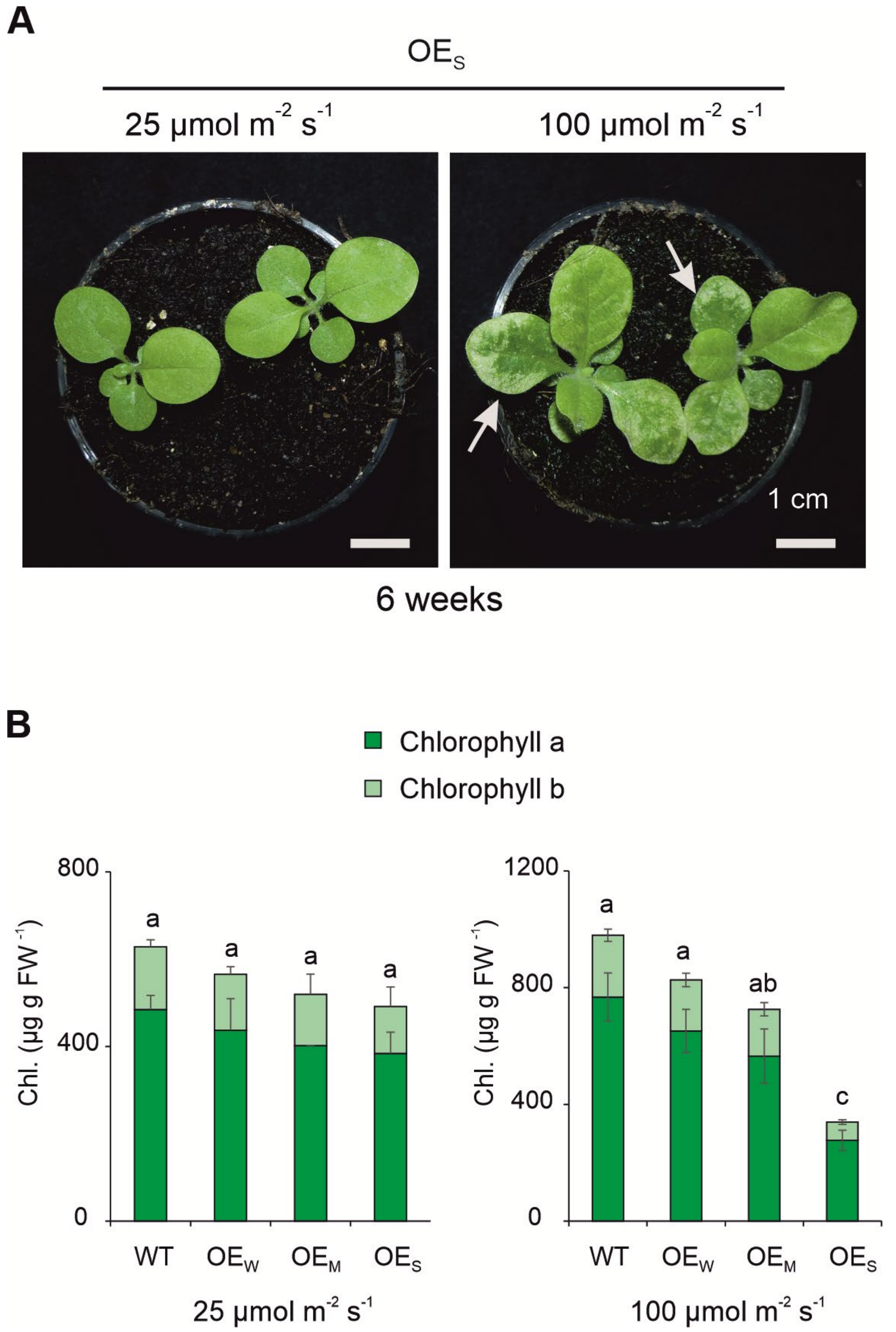

Supplementary Figure S2. Comparison of OE$_S$ plants grown under 25 µmol photons m^{-2} s^{-1} or 100 µmol photons m^{-2} s^{-1} light intensity. (Supports **Figure 2**)

(**A**) Phenotypes of OE$_S$ plants grown under 25 µmol photons m^{-2} s^{-1} or 100 µmol photons m^{-2} s^{-1} for 6 weeks after seed germination. White arrows point to necrotic sectors appearing upon growth at 100 µmol photons m^{-2} s^{-1}. (**B**) Chlorophyll contents of plants grown under 25 µmol photons m^{-2} s^{-1} (left diagram) and 100 µmol photons m^{-2} s^{-1} (right diagram). Values represent means ± SD of three independent biological replicates. Lowercase letters indicate statistically significant differences between mean values among different genotypes ($P < 0.05$, one-way

ANOVA with Tukey's honestly significant difference [HSD] post hoc test). OE_W: weak overexpressing line, OE_M: medium overexpressing line, OE_S: strong overexpressing line.

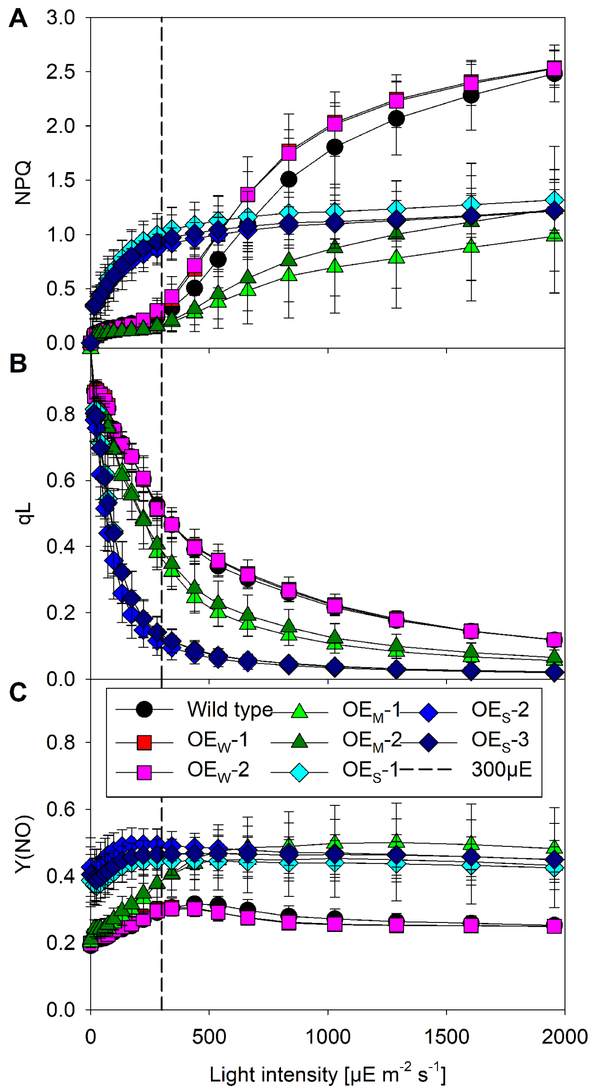

Supplementary Figure S3. Photosynthetic parameters in the wild type and cytochrome *b*559 overexpression plants. Light response curves of the chlorophyll-*a* fluorescence parameters (**A**) non-photochemical quenching (NPQ), (**B**) qL (a measure for the redox state of the PSII acceptor side), and (**C**) Y(NO), a measure for the non-regulated dissipation of excitation energy by PSII, are shown. (Supports **Table 1**)

The dotted vertical line indicates the growth light intensity of 300 µmol photons m^{-2} s^{-1}. For the wild type and each independent mutant line, average values with standard deviation are shown (n = 12). OE_W: weak overexpressing lines, OE_M: medium overexpressing lines, OE_S: strong overexpressing lines.

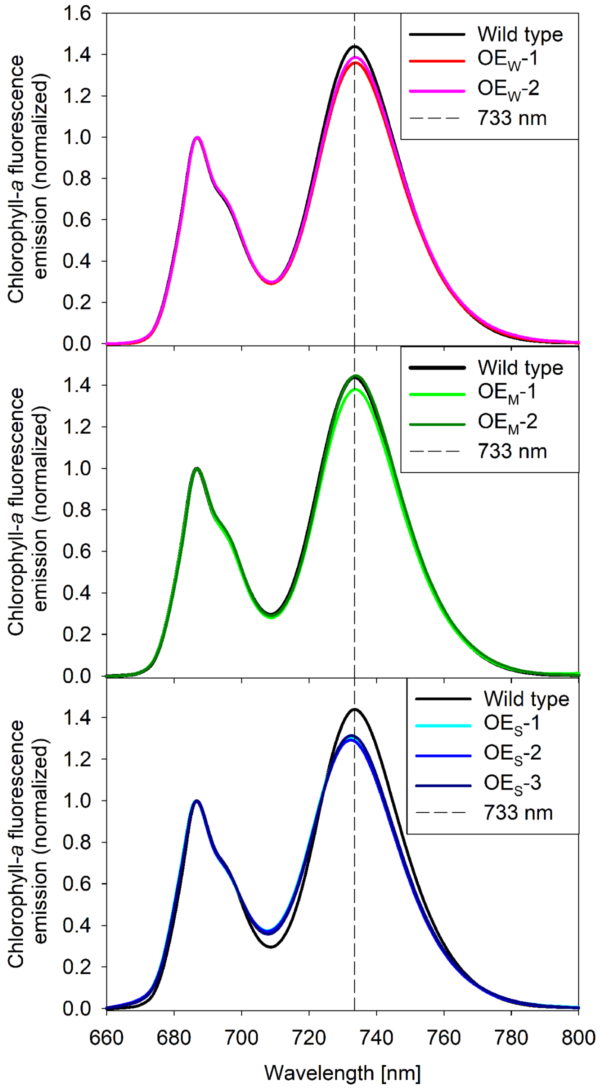

Supplementary Figure S4. 77K chlorophyll-*a* fluorescence emission spectra of the wild type, two independent OE_W and OE_M lines each, and three independent OE_S lines. (Supports **Table 1**)

The emission spectra were normalized to the emission maximum of PSII (photosystem II) at 685 nm wavelength. The dotted vertical line indicates the emission maximum of PSI-LHCI (photosystem I with light-harvesting complex I). The shifted emission maximum of PSI and increased emission between 710 and 725 nm wavelength in OE_S indicates the presence of free, uncoupled LHCI (n = 6 for all genotypes). OE_W: weak overexpressing lines, OE_M: medium overexpressing lines, OE_S: strong overexpressing lines.

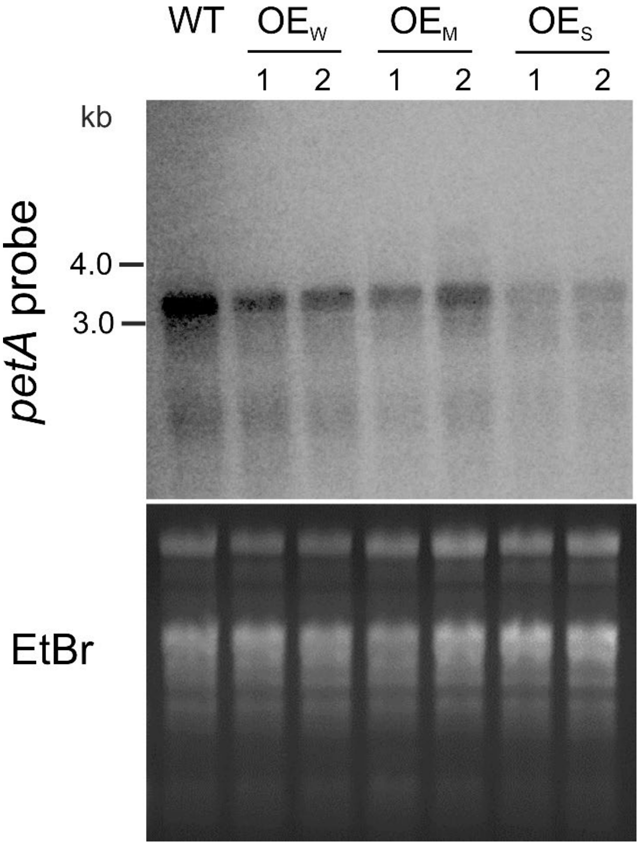

Supplementary Figure S5. Analysis of *petA* mRNA accumulation in OE$_W$, OE$_M$ and OE$_S$ transplastomic plants in comparison to wild-type control plants (WT). (Supports **Figure 3**)
Samples of 3 µg extracted total cellular RNA were electrophoretically separated in a 1% denaturing agarose gel and hybridized to a radiolabeled *petA* probe generated by PCR amplification with gene-specific primers (Supplementary Table S1). Two independently generated transplastomic lines for each construct were analyzed. To control for equal loading, the ethidium bromide (EtBr)-stained agarose gel was photographed prior to blotting, and is shown below the blot. Note the decreased *petA* mRNA accumulation in the transplastomic lines due to the antisense RNA effect resulting from transcriptional overexpression of the *psbE* operon (Ghandour et al., 2023). See text for details. OE$_W$: weak overexpressing lines, OE$_M$: medium overexpressing lines, OE$_S$: strong overexpressing lines.

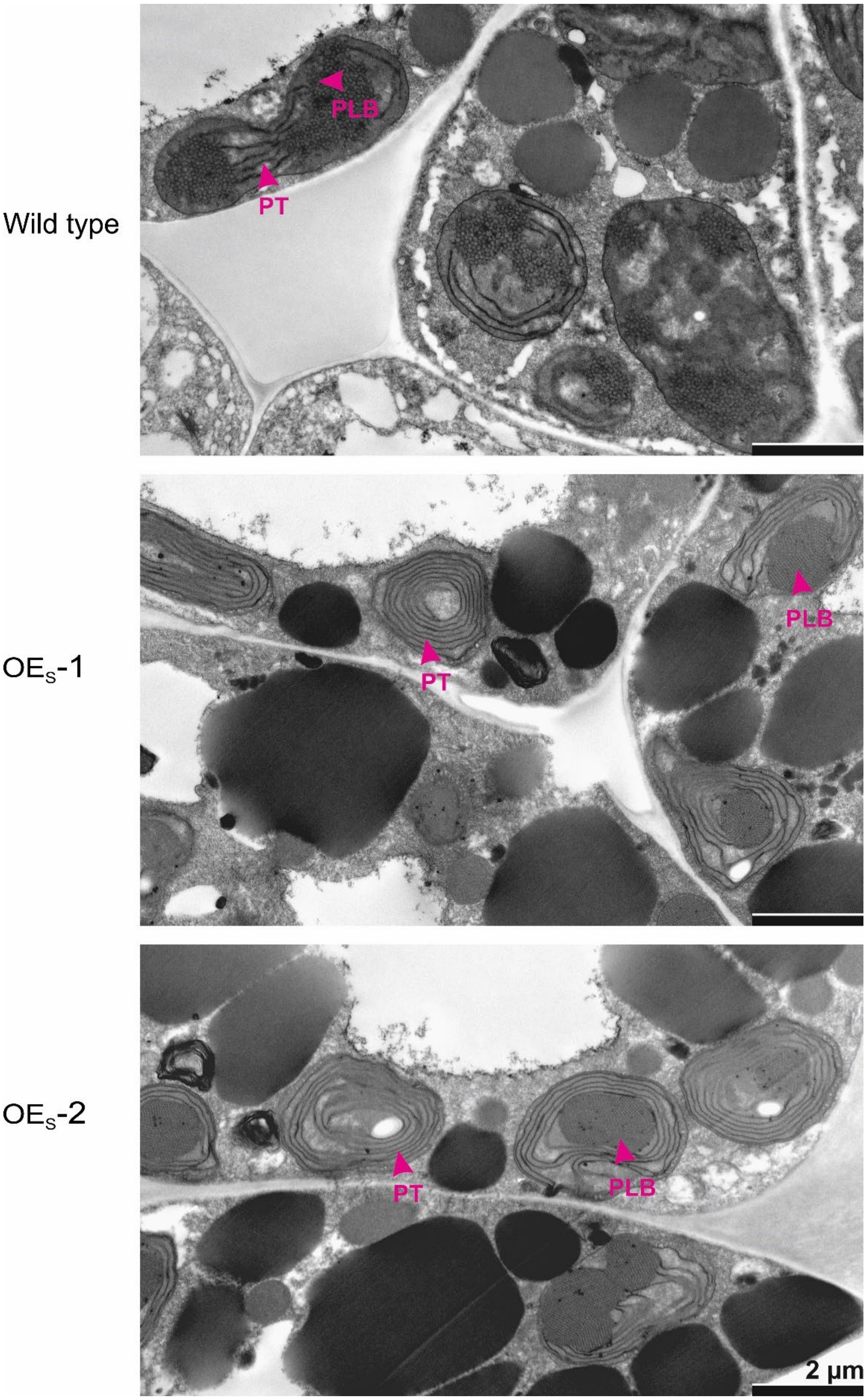

Supplementary Figure S6. Plastid ultrastructure in cotyledons of 7-day-old etiolated wild type and OE_S seedlings. (Supports **Figure 5**)

The TEM (transmission electron microscopy) analysis shows representative etioplasts in the wild type, and the OE_S-1 and OE_S-2 transplastomic lines. PLB: prolamellar body; PT: prothylakoid. OE_S: strong overexpressing lines.

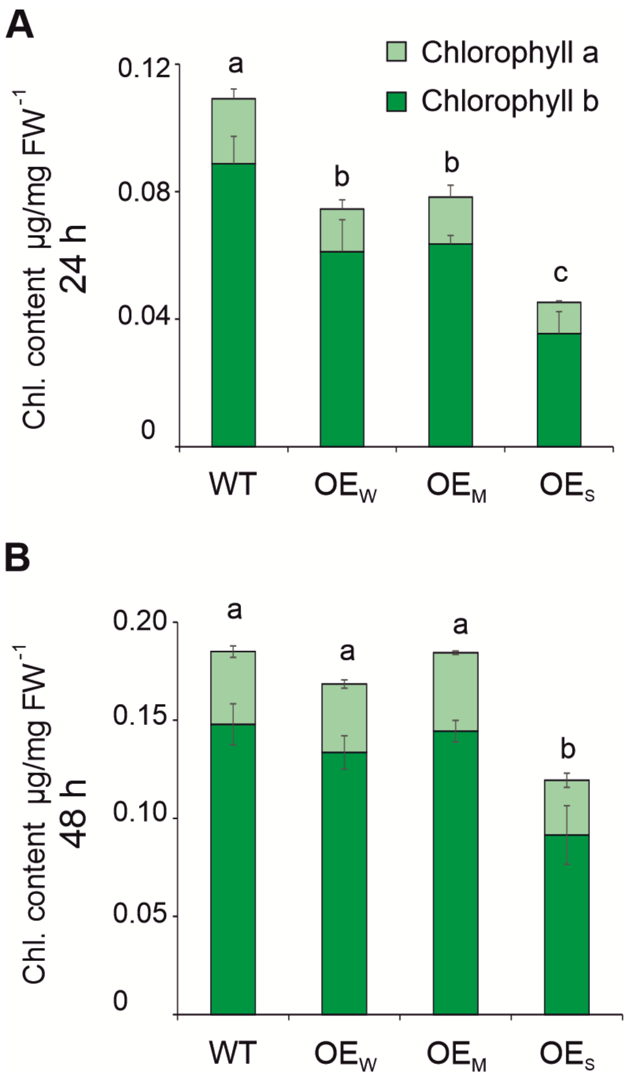

Supplementary Figure S7. Increase in chlorophyll contents in the wild type (WT) and the transplastomic OE_W, OE_M and OE_S plants during deetiolation. (Supports **Figure 6**)

(**A**) Chlorophyll contents after 24 h of illumination. Values represent means ± SD of at least three independent biological replicates. Lowercase letters indicate statistically significant differences between mean values among different genotypes ($P < 0.05$, one-way ANOVA with Tukey's post hoc honestly significant difference [HSD] test). (**B**) Chlorophyll contents after 48 h of light exposure. Values represent means ± SD of at least three independent biological replicates. Lowercase letters indicate statistically significant differences between mean values among different genotypes ($P < 0.05$, one-way ANOVA with Tukey's post hoc honestly significant difference [HSD] test). OE_W: weak overexpressing line, OE_M: medium overexpressing line, OE_S: strong overexpressing line.

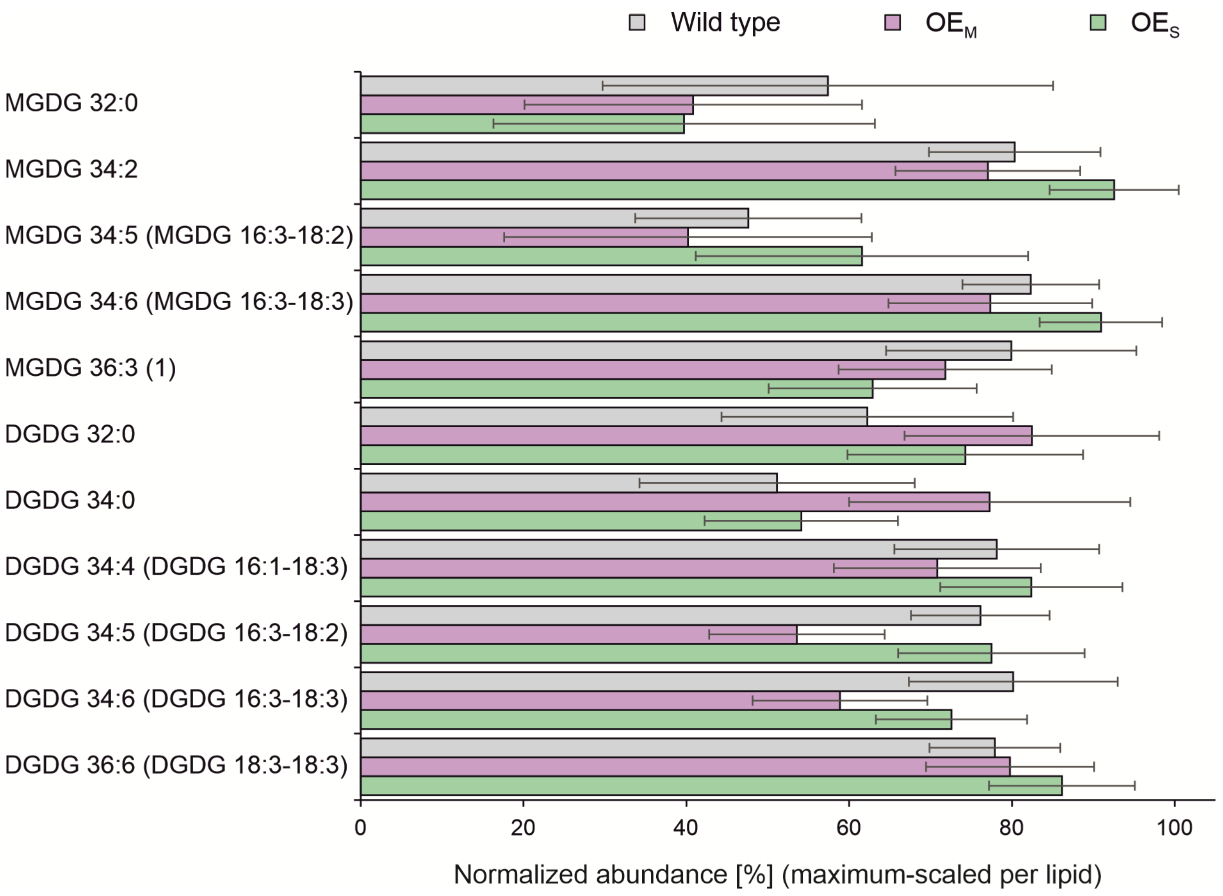

Supplementary Figure S8. Galactolipids with largely unaltered accumulation in etiolated cotyledons of the OE$_S$ and OE$_M$ transplastomic plants relative to the wild type. (Supports **Figure 7**)

Lipid abundances were quantified by lipidomic profiling using liquid chromatography-tandem mass spectrometry with normalization to sample fresh weight and the internal standard PC 34:0 (PC 17:0-17:0). Lipid abundances are maximum-scaled to allow for comparison of abundant and minor lipid species. Unless verified by authenticated reference lipids, lipid species are named by lipid class, sum of carbon atoms in acyl chains and degree of unsaturation. Chromatographically separated structural isomers of lipids are indicated by numbers in parentheses. Data are means ± SD of 5 to 12 independent biological replicates each (see Methods for details). Note the enrichment of galactolipid species from the prokaryotic biosynthesis pathway. OE$_M$: medium overexpressing line, OE$_S$: strong overexpressing line.

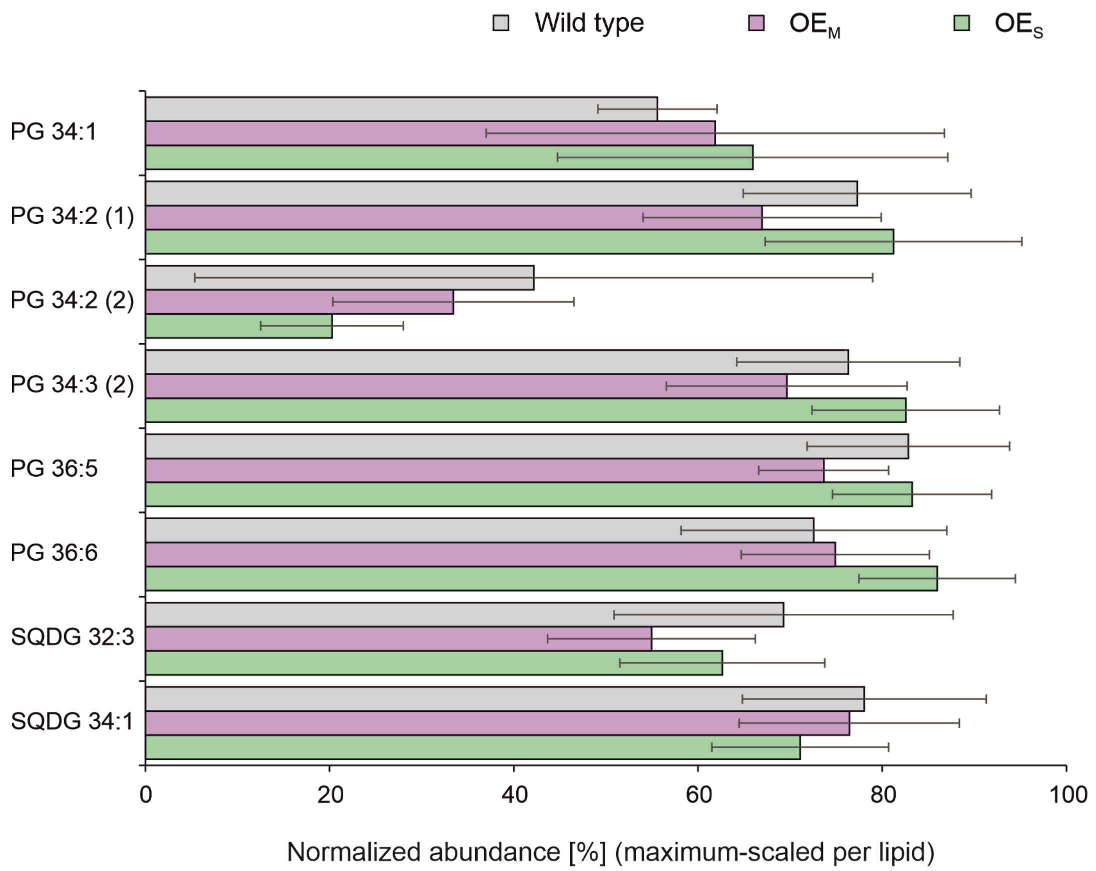

Supplementary Figure S9. Anionic plastid lipids (PG and SQDG) with largely unaltered accumulation in etiolated cotyledons of the OE$_S$ and OE$_M$ transplastomic plants relative to the wild type. (Supports **Figure 8**)

Lipid abundances were quantified by lipidomic profiling using liquid chromatography-tandem mass spectrometry with normalization to sample fresh weight and the internal standard PC 34:0 (PC 17:0-17:0). Lipid abundances are maximum-scaled to allow for comparison of abundant and minor lipid species. Lipid species are named by lipid class, sum of carbon atoms in acyl chains and degree of unsaturation. Chromatographically separated structural isomers of lipids are indicated by numbers in parentheses. Data are means ± SD of 5 to 12 independent biological replicates each (see Methods for details; Student´s t-test $P > 0.05$). OE$_M$: medium overexpressing line, OE$_S$: strong overexpressing line.

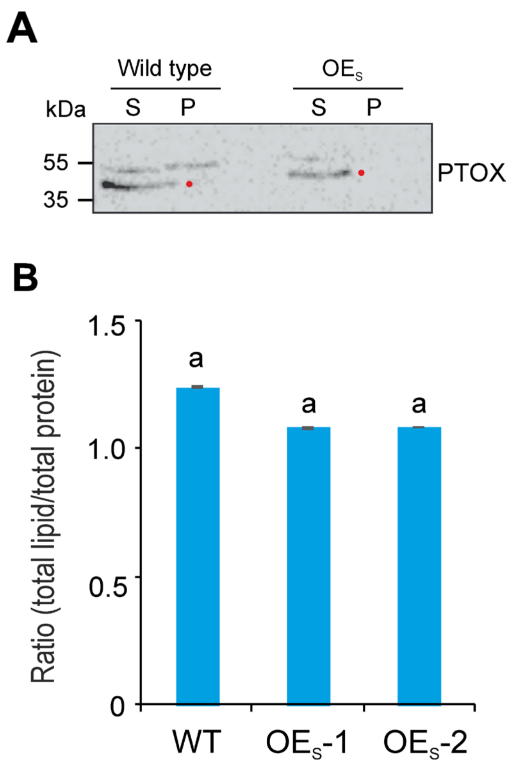

Supplementary Figure S10. Determination of PTOX protein amounts, and total lipid/total protein ratios in wild type and OE$_S$ plants. (Supports **Figure 9**)

(**A**) Samples of 100 µg total cellular protein were separated into soluble (S) and pellet (P) fractions, electrophoretically separated by 12% SDS-PAGE (polyacrylamide gel electrophoresis), blotted, and immunodecorated with PTOX-specific antibodies. The red dots mark the specific PTOX signals, signals of larger size represent a non-specific cross-reaction (https://www.agrisera.com/en/artiklar/ptox-plastid-terminal-oxidase.html). **(B)** Determination of total lipid/total protein ratios in etiolated wild type and OE$_S$ seedlings. Total lipids were determined by a colorimetry-based method (see Methods section). Total protein amounts were determined by the bicinchoninic acid assay (Pierce™ BCA Protein Assay Kits). Values were calculated based on the average of total lipids from three biological replicates from the wild type (WT), and two biological replicates from OE$_S$-1 and OE$_S$-2, respectively, divided by total protein amounts acquired from the average of three biological replicates from the wild type and the two OE$_S$ lines. Data were obtained from extraction of 30 mg (fresh weight) seedlings of each genotype. Error bars represent the standard deviation (SD). No statistically significant differences between the three genotypes were obtained ($P > 0.05$, one-way ANOVA with Tukey's post hoc honestly significant difference [HSD] test). OE$_S$: strong overexpressing lines.

SUPPLEMENTARY TABLES

Supplementary Table S1. List of oligonucleotides used as PCR primers in this study.

Primer name	Target	Sequence 5' → 3'
oBQ101	*ACTIN*	TCACAGAAGCTCCTCCTAATCC
oBQ102	*ACTIN*	GGGAAAGAACAGCCTGAATG
oBQ103	*APXc*	TGTTCCCTTTCACCCTGGTAGAG
oBQ104	*APXc*	CGTTCCTTGTGGCACCTTCC
oBQ105	*CAT1*	CGCCATGCTGAGAAGTATCC
oBQ106	*CAT1*	AAAGCGTTCTTGCCTGTCTG
oBQ123	*BAP1*	AGGGTGAGAAGAACGGGATTA
oBQ124	*BAP1*	CGTCCACTGCTGCGAATAA
oBQ125	*ZAT12*	CCACCGTGCAAGTCATAAAC
oBQ126	*ZAT12*	GACCCAAAGAGAACTCCATACC
oBQ127	*WRKY40*	TGGGCTTGGAATAGATCACAC
oBQ128	*WRKY40*	TAACATCTCCGTCAGCTTCTTG
oTAM3	*aadA*	GGATCCAAGAAAAGTGAGC
oTAM2	*aadA*	CTAGTGGATCGCACTCTACC
oTAM1	*aadA_Prrn*	GGATCCAAGAAAAGTGAGC
oTAM4	*Prrn_T7g10*	GAAATTGAGCTCGCTC
oTAM5	*Prrn_T7g10*	ATCTCCTTCTTAAAGTTAAACA
oTAM6	*psbE_psbF*	GGATGAACTGCATTGC
oTAM7	*psbE_psbF*	CACAGGAGAACGTTCG
oTAM99	*psbE*	CTAAAACGATCTACTAAATTCATCG
oTAM101	*psbE*	ATGTCTGGAAGCACAGGAG
oBQ170	*petA*	GGTACTTCCTGATACTGTATTTGAA
oBQ171	*petA*	CATCCGACGCATCCGTTATG

Supplementary Table S2. List of antibodies used in this study.

Antibody	Generated in	Dilution used	Source
PsbE	Rabbit	1:5000	Agrisera
PsbD	Rabbit	1:8000	Agrisera
PetA	Rabbit	1:2000	Agrisera
PetB	Rabbit	1:5000	Agrisera
PsaF	Rabbit	1:1000	Agrisera
PsaH	Rabbit	1:1000	Agrisera
PsaB	Rabbit	1:1000	Agrisera
AtpB	Rabbit	1:5000	Agrisera
POR	Rabbit	1:5000	Agrisera
PTOX	Rabbit	1:4000	Agrisera
