# Supplementary material for: Membrane protein provision controls prothylakoid biogenesis in tobacco etioplasts
Source: Plant Cell. 2024 Sep 25;36(12):4862–80. doi: 10.1093/plcell/koae259 (PMC11638105; doi:10.1093/plcell/koae259)
Supplement: koae259_Supplementary_Data [file tpc.01016.2023-s12.pdf › tpc.01016.2023-s12.pdf]

THE PLANT CELL

Control of prothylakoid biogenesis by membrane protein provision in tobacco etioplasts

Bingqi Li, Tegan Armarego-Marriott, Łucja Kowalewska, Wolfram Thiele, Alexander Erban, Stephanie Ruf, Joachim Kopka, Mark Aurel Schöttler, and Ralph Bock

Review Timeline:

Submission Date:	18-Sep-2023
Editorial Decision:	18-Oct-2023
Resubmission Received:	10-Jul-2024
Editorial Decision:	30-Jul-2024
Revision Received:	12-Aug-2024
Accepted:	13-Aug-2024

Prof. Dr. Ralph Bock
Max-Planck-Institut fur molekulare Pflanzenphysiologie
Dept. III
D-14476 Potsdam-Golm
Germany

Dear Dr. Bock/Dear Ralph:

Thank you for choosing to send your manuscript entitled "Control of prothylakoid biogenesis by membrane protein provision in tobacco etioplasts" for consideration at The Plant Cell. Your submission has been evaluated by members of the editorial board as well as expert reviewers in your field, and we regret to inform you that we are not able to recommend the publication of this manuscript in its current form. We have had input from multiple scientists and we have solicited post-review comments.

During the post-review consultation session, we also agreed that if you could address the major points raised by the reviewers by new experiments, we would welcome a resubmission. This may be treated as a new submission, but we would attempt to use at least some of the same reviewers. Nevertheless, reviewers will be asked to assess as a new manuscript (i.e. are the claims fully supported by the data and do the results presented move the field forward?), and not only whether previous reviewer comments have been addressed.

While the reviewers praise the high quality of the work and the breadth of the techniques used, and recognize the significant interest of the topic, they also express concerns about the conclusions drawn from the current data. As detailed in their comments, all reviewers believe that additional data are required to substantiate the conclusions about the role of cytb-559. Specifically, a thorough analysis of PsbF and heme is crucial to link the current results to Cyt b-559. Additionally, the overexpression of at least one other protein is necessary to support the specific role of PsbE in thylakoid proliferation.

We thank you for your interest in and support of The Plant Cell. We hope we will be able to render a more positive decision on future work.

Sincerely,

The Plant Cell Board of Editors

-- Reviewer comments:

Reviewer #1 (Comments for the Author):

Importance of findings:
Cyt b-559 is known to play a physiologically important role in the assembly and maintenance of PSII in mature chloroplasts but the molecular mechanisms are still unclear and it remains quite possible that Cyt b-559 has functions outside PSII. The current submission links over-expression of Cyt b-559 to the proliferation of prothylakoids in etioplasts. This is a most interesting finding and will be of general interest to the readership of The Plant Cell.

Quality of experiments:
In general, a wide range of experiments have been performed and the data are of high quality with appropriate statistics.

Points in favour:
The novelty of the phenotype and wide range of experimental techniques used.

Points detracting:

1. The authors state that levels of Cyt b-559 have increased by a factor of 10 in etioplasts of the OE_S plants. This is based on the data in Fig 4. It would be preferable to show a dilution series of OE_S compared to WT signal so that the signal falls within the WT range.
2. The authors have confirmed that levels of PsbE have increased in etioplasts but have not confirmed whether PsbF levels are also elevated and whether haem is bound. Some spectroscopic data supporting the formation of Cyt b-559 would seem necessary here.
3. The authors explain the 'prothylakoid snailing' phenotype on the increased protein abundance of Cyt b-559. However, it remains possible that Cyt b-559 might be enzymatically active in the etioplast and be involved in the oxidation of plastoquinol

(and possibly tocopherol) and so play a role in controlling the redox state of the PQ pool, with potential impacts on redox signalling and redox control of transcription. Perhaps this could be discussed. Have the authors done a global analysis of plastome transcript levels to see if there are any additional effects on transcription?
4. The authors confirmed in Fig 3 enhanced transcript levels of the psbEFLJ operon but fail to determine whether levels of PsbL and PsbJ (and PsbF) are likewise elevated. Consequently, it seems premature to conclude that the effect on membrane ultrastructure is due to expression of Cyt b-559. Possibly this could be addressed by overexpressing psbE and psbF at a neutral site.
5. Fig 4: it would be useful to know whether the expression of NDH and PTOX, known to be expressed in etioplasts, have been affected in the mutants, for which antibodies are available. Also, have the authors attempted to conduct a more wide-ranging proteomic analysis on the prothylakoids?
6. Have the authors assessed whether overexpressed PsbE is associated with other proteins/complexes?
7. Have transcript levels of psbEFLJ been determined for etioplasts? Fig 3 seems to be for light-grown plants.
8. The title and abstract emphasize the possibility that the abundance of membrane proteins regulates lipid biosynthesis. If the authors believe it to be a general phenomenon, then the authors could over-express a second target or analyse existing mutants (of Cyt b6f for example) where a protein has been overexpressed (Rieske FeS protein?). Otherwise, the authors might like to modify the text appropriately.
9. The authors suggest that protein synthesis is coordinated to lipid biosynthesis. If this is the case, one would expect the lipid/protein ratio to be constant in WT and mutants. Has this been determined?
10. The authors suggest that over-expression of Cyt b-559 affects PSII function during greening leading to ROS production. Have the authors any clues on the mechanism? Could PSII repair be affected because of reduced D1 synthesis? Is there impaired synchronization in the accumulation of PSI and PSII?
11. The authors mention in the discussion that Cyt b-559 is a nucleation factor for PSII assembly and its presence in the prothylakoids could speed up the greening process. Yet, this seems to be the opposite of what is observed in the mutant. Some discussion needed.

Author actions necessary for acceptance
1. Confirmation that Cyt b-559 is formed and is present at elevated levels
2. PTOX and NDH blots
3. Determination of protein/lipid ratios
4. Analysis of a second mutant in which a thylakoid protein has been over-expressed to test whether the phenotype is specific to psbEFLJ operon.

Reviewer #2 (Comments for the Author):

Li and colleagues report engineering plastid "cytochrome b559 overexpressor tobacco plants", with the goal of understanding the role of the cytochrome in etiolated seedlings, where it accumulates in the WT context despite the absence of Photosystem II. b559 is a cytochrome of elusive function, while being part of the very core of Photosystem II across photosynthetic phyla. The premise the authors base their research on is interesting, and the question of the PSII assembly and function timely. I have, however, strong reservations about the possibility of providing conclusions that the authors propose within their experimental framework.

Given that the Photosystem II is degraded in the majority of mutants (including point mutations) of cyt b559, the authors design three overexpressor lines. They modified the chloroplast genome to increase the at both transcriptional and translational levels, acting on upstream elements of the entire psbE/F operon, and specifically on the 5' UTR of the first gene in the polycistronic product, psbE. The design and verification of these constructs and three overexpressor lines are solid, and include immunoblots against the gene products and other photosynthetic complexes, as well as northern blots to verify the transcript levels.

The authors report tenfold increase in the PsbE accumulation than in the WT (although it looks more like a hundred-fold difference). Unfortunately, they do not report on the PsbF subunit of the apocytochrome, the first of three major experimental flaws of the paper, that in my opinion does not permit to draw the conclusions proposed by the authors. At that point, the article changes toward explanation of the bizarre phenotype observed in the PsbE overexpressing transplastomic plant, and despite essentially describing PsbE overexpressor continues to label the figures "strong cytochrome b559 overexpressor plants...". This is highly misleading, the holocytochrome b559 is comprised of PsbE, PsbF and the haem: neither of the latter two are present in increased amounts in the plants (or their presence was not verified). It is highly possible that even if PsbF was somehow upregulated in the OEs plants, the haem amount would be limiting for the accumulation of the holocytochrome, as no effort of boosting haem synthesis was made in these plants. The lack of PsbF co-overexpression, or haem synthesis enhancement - necessary steps in the process of "cytochrome b559 overexpression" - make it not acceptable to use such nomenclature in this manuscript, or draw conclusions about the cytochrome b559 role. Similarly, the discussion section states that the authors tried to address the function of cytochrome b559 by pursuing an overexpression approach - which is false, as in the best-case scenario they have attempted to overexpress two subunits of the apocytochrome.

Furthermore, the overexpression misses at least one control: assuming that in etiolated OEs plants the PsbF level, as well as

available haem levels follow the PsbE amount, reporting on any functional consequences of the cytochrome in, e.g., photoprotection requires a point mutant of one of the subunits of the cytochrome where the haem function is altered without a decrease of the PSII assembly and accumulation. This might be challenging given the previous unsuccessful trials of b559 mutagenesis.
However, the authors also propose that PsbE (and not as they write "cyt. b559") controls, or exhibits important role in the deetionlation and/or PSII assembly. The evidence for that is the slightly lower rate of greening, and a membrane ultrastructure differences in the condition of very strong hydrophobic helix overaccumulation. This kind of claim clearly calls for an overexpression of other similar proteins to a similar extent, as the simple interpretation of the phenotype suggest that a mere presence of any excess hydrophobic molecule will result in an imbalance in proteostasis/membrane integrity. In other words, the link between the *specific* role of PsbE (let alone cyt. b559) is very much missing. It is indeed likely that the last sentence of the results section best encapsulates the findings (but needs a much stronger support that what is provided by the Authors): "...the abundance of membrane proteins in etioplasts limits prothylakoid biosynthesis, in that prothylakoids are continuously made until all synthesized proteins have been accommodated". This - rather than a specific role of PsbE (and even less so the holocytochrome b559) is what I suggest the authors try to prove.

Reviewer #3 (Comments for the Author):

The authors of this paper pose the enigmatic question about the role of cyt b559 in PSII, which "has defied all attempts to elucidate its precise function in PSII function and biogenesis". Here, the authors focus on their structural role - in particular, during the biogenesis in angiosperms (here, tobacco) that do not synthesize chl in the dark but their etioplast / prolamellar body (PLB) contains cyt b559 but no PSII. Via generating psbE and psbF overexpressing (OE) tobacco, the authors provide evidence for a structural role of cyt b559 in PLB formation and prothylakoid biogenesis; their data also *strongly suggest* that OE of thylakoid membrane proteins regulates lipid biosynthesis in etioplasts. I think, these are important and valid conclusions - but with limitations, which should be clarified by the authors; also some corrections / amendments are requested.

1/ Although the authors - correctly and carefully - draw their conclusions, I think for the 'general reader of TPC' it would be useful to emphasize that:

(i) These data do not give us the key to understand the structural and functional roles of cyt b559 in PSII, in general (e.g. in systems that do not synthesize PLB, or yet alone in cyanobacteria). I think considerations on these issues would be useful and some refinment of the text would help. For instance, the first sentence in Discussion may leave the false and evidently unintended impression that the authors obtained information about any particular structural/functional role of cyt b559. It can easily be envisioned that OE of other proteins present in etioplasts would lead to very similar effects. Also, as above, conclusions reached in the case of PLB and PTs won't explain the role of cyt b559 e.g. in cyanobacterial PSII. This should be clarified.

(ii) Similarly, it remains to be answered or at least commented how much the effect of overexpression of cyt b559 might compare with OE of other membrane proteins. It is very likely but I do not see supporting data allowing us to conclude that "the abundance of thylakoid membrane proteins in etioplasts regulates
lipid biosynthesis and, in this way, controls prothylakoid biogenesis". (I fully agree with the authors that "regulation of membrane biogenesis by protein provision could be a more general theme in the biosynthesis of biological membrane systems.)

2/ Additional comments:

(i) Fv/Fm cannot and should not be equated with the maximum quantum efficiency of PSII - see Garab et al. 2023 JXB (and references therein). A more cautious and detailed analysis of ChlF is requested; the raw ChlF data would probably provide explanation on the nature of changes in PSII that lead to the observed decrease of Fv/Fm. E.g., to the present reviewer it appears very likely that OE of cyt b559 may lead to the accummulation of not fully matured PSII, with all - pleiotropic consequences (as in ll. 371-373). Thus, in the simplest case, instead of suggesting a decreased quantum efficiency, a mixture of active and inactive(d) PSII RCs might offer at least a phenomenologically correct explanation.

(ii) l. 292: "PSII is the major site of 1O2 generation in the chloroplast" -a more detailed explanation is required. I presume the authors refer to the specific conditions of 1O2 generation via the formation of chl triplets. Please refine this section.

(iii) In several cases (e.g. l. 311) the authors fail to add that significant increases are given relative to what.

Point-by-point response to the Reviewers' suggestions (with our response in blue)

Reviewer #1 (Comments for the Author):

Importance of findings:
Cyt b-559 is known to play a physiologically important role in the assembly and maintenance of PSII in mature chloroplasts but the molecular mechanisms are still unclear and it remains quite possible that Cyt b-559 has functions outside PSII. The current submission links over-expression of Cyt b-559 to the proliferation of prothylakoids in etioplasts. This is a most interesting finding and will be of general interest to the readership of The Plant Cell.

Quality of experiments:
In general, a wide range of experiments have been performed and the data are of high quality with appropriate statistics.

Points in favour:
The novelty of the phenotype and wide range of experimental techniques used.

Points detracting:
1. The authors state that levels of Cyt b-559 have increased by a factor of 10 in etioplasts of the OE_S plants. This is based on the data in Fig 4. It would be preferable to show a dilution series of OE_S compared to WT signal so that the signal falls within the WT range.
We agree and have included dilution series of both the wild-type samples, and the OE_S samples in an additional immunoblot (Figure 4B). The data confirmed an approximately tenfold overexpression of PsbE.

2. The authors have confirmed that levels of PsbE have increased in etioplasts but have not confirmed whether PsbF levels are also elevated and whether haem is bound. Some spectroscopic data supporting the formation of Cyt b-559 would seem necessary here.
To provide these data, we produced very large amounts of etiolated plant material to be able to isolate sufficient amounts of intact etioplasts from wild type and OE_S plants for spectroscopic quantification of the cytochrome. The quantification revealed a 1.44-fold increase of Cyt b_{559} in OE_S plants. The data are included in Table 1 and discussed in the text on pp. 9-10 of the revised manuscript.

3. The authors explain the 'prothylakoid snailing' phenotype on the increased protein abundance of Cyt b-559. However, it remains possible that Cyt b-559 might be enzymatically active in the etioplast and be involved in the oxidation of plastoquinol (and possibly tocopherol) and so play a role in controlling the redox state of the PQ pool, with potential impacts on redox signalling and redox control of transcription. Perhaps this could be discussed. Have the authors done a global analysis of plastome transcript levels to see if there are any additional effects on transcription?
We thank the reviewer for this suggestion, and we now discuss this possibility (p.15 of the revised ms). To assess possible effects on the redox state of the PQ pool through PTOX, we have also tested PTOX protein accumulation in etiolated wild type and OE_S seedlings, and found it to be unaltered (Supplementary Figure 10A), suggesting that PTOX accumulates largely independently of b_{559}.

4. The authors confirmed in Fig 3 enhanced transcript levels of the psbEFLJ operon but fail to determine whether levels of PsbL and PsbJ (and PsbF) are likewise elevated. Consequently, it

seems premature to conclude that the effect on membrane ultrastructure is due to expression of Cyt b-559. Possibly this could be addressed by overexpressing psbE and psbF at a neutral site.
We have determined *psbE* operon transcript levels (shown in Figs. 3 and 4), and the transcript comprises all four genes of the operon: *psbE*, *psbF*, *psbL* and *psbJ*. Thus, at the mRNA level, all four genes are equally strongly overexpressed. We explain this in the main text, by stating that the 1.1 kb operon transcript comprises all four reading frames and does not undergo post-transcriptional cleavage into monocistronic mRNAs (p. 4), also providing appropriate references. Overexpression of *pbsE* and/or *psbF* at a neutral site is, unfortunately, not possible, because due to the high homologous recombination activity in the chloroplast, the ectopic copy of the gene(s) would undergo recombination with the resident copy, thus causing rearrangements (deletions) in the chloroplast genome.

5. Fig 4: it would be useful to know whether the expression of NDH and PTOX, known to be expressed in etioplasts, have been affected in the mutants, for which antibodies are available. Also, have the authors attempted to conduct a more wide-ranging proteomic analysis on the prothylakoids?
See point 3. As suggested, we have analyzed PTOX accumulation by immunoblot analysis of etiolated wild type and OE_S seedlings (Supplementary Figure 10A), and no alteration was detected. Unfortunately, the NDH antibodies did not work in total protein extracts from etiolated seedlings – at least in our hands, the antibodies work only in isolated thylakoid membranes. Prothylakoid proteomics will be an interesting approach for our future studies, but will require large amounts of etiolated material and thorough optimization of membrane isolation protocols.

6. Have the authors assessed whether overexpressed PsbE is associated with other proteins/complexes?
At this point, we have no evidence for association of the overaccumulating PsbE with other protein complexes. The new data added to our revised ms show that only part of the overexpressed PsbE forms active cytochrome b_{559}. Whether or not the unassembled fraction is solely floating within the prothylakoid membrane or, in part, associating with other proteins, we currently do not know. This could potentially be addressed by expressing an epitope-tagged version of PsbE for co-immunoprecipitation, but the approach would bear the risk that the tag interferes with structure, function or membrane integration of the (small) PsbE protein.

7. Have transcript levels of psbEFLJ been determined for etioplasts? Fig 3 seems to be for light-grown plants.
We have performed additional RNA gel blots to answer this question, and the results are presented in the new Fig. 4C. The data confirm that the 1.1 kb tetracistronic *psbE* operon transcript is also the major RNA species accumulating in etiolated OE_S seedlings, and reveal that the overexpression level is lower in etioplasts than in chloroplasts (although the oeverexpression at the protein level is much higher). This is discussed on p. 10 of our revised ms in the context of the stability of the overexpressed protein in chloroplasts vs. etioplasts.

8. The title and abstract emphasize the possibility that the abundance of membrane proteins regulates lipid biosynthesis. If the authors believe it to be a general phenomenon, then the authors could over-express a second target or analyse existing mutants (of Cyt b6f for example) where a protein has been overexpressed (Rieske FeS protein?). Otherwise, the authors might like to modify the text appropriately.

This is a good point, which however, cannot be easily addressed experimentally. We explain this in our revised manuscript as follows (p.15-16): "It would be interesting to overexpress other thylakoid membrane proteins in etioplasts and analyze the consequences at the ultrastructural level. However, our efforts to achieve overexpression of other thylakoid proteins have been unsuccessful. This is unsurprising, given that cytochrome b_{559} is the only small protein subcomplex known to accumulate in etioplasts. Although the NADH dehydrogenase complex, the ATP synthase and the cytochrome b_6f complex also accumulate in the dark, the individual subunits of these large complexes are not stable unless faithfully assembled into the complexes (e.g., Shikanai et al., 1998; Hager et al., 1999). Thus, overexpression of most, if not all, subunits of one of these complexes is likely required to obtain another case of prothylakoid membrane snailing."
Two examples of our efforts are shown in the figure below (attempted overexpression of the b6f subunits PetA and PetN). As can be seen from panel A, no significant overexpression could be achieved (likely due to instability of the unassembled overexpressed subunits), and consequently, no thylakoid snailing is observed (panel B).

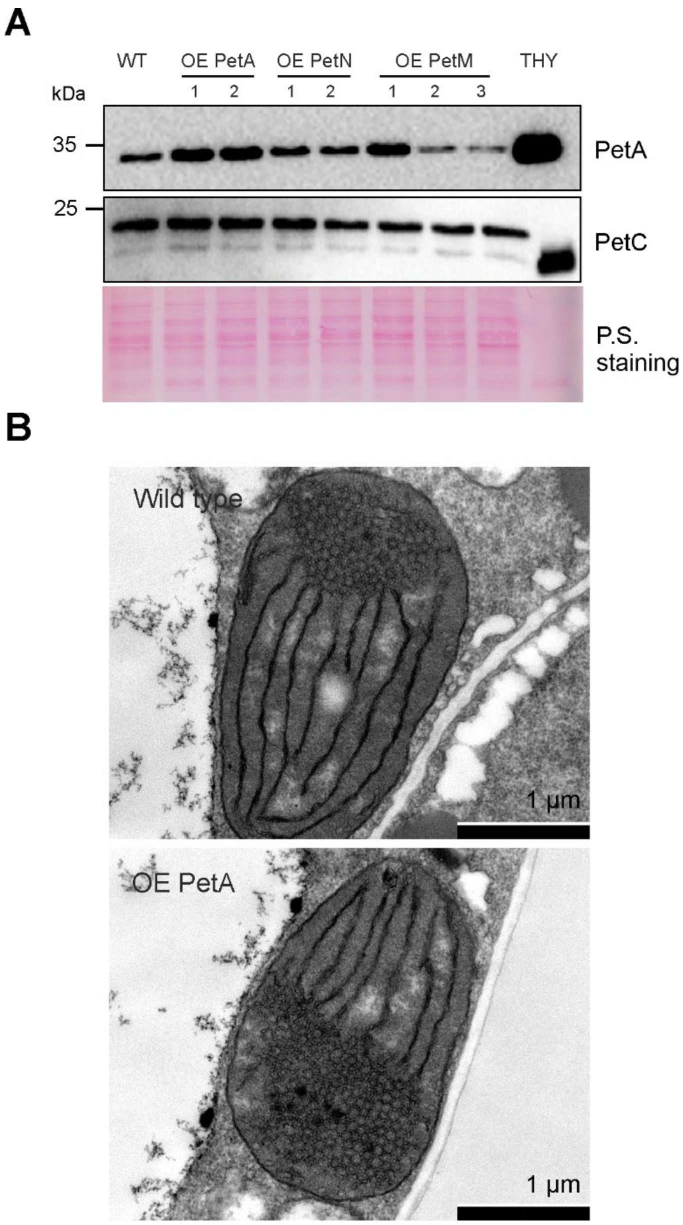

9. The authors suggest that protein synthesis is coordinated to lipid biosynthesis. If this is the case, one would expect the lipid/protein ratio to be constant in WT and mutants. Has this been determined?
As suggested by the Reviewer, we have determined the lipid/protein ratios, and found them to be not significantly altered. The data are shown in the new Supplemental Figure 10.

10. The authors suggest that over-expression of Cyt b-559 affects PSII function during greening leading to ROS production. Have the authors any clues on the mechanism? Could PSII repair be affected because of reduced D1 synthesis? Is there impaired synchronization in the accumulation of PSI and PSII?
To better understand the increased susceptibility of the OES mutants to photooxidative damage, we have measured a number of additional parameters, including light response curves of several chlorophyll-*a* fluorescence parameters (new Supplemental Fig. 3), and also recorded 77K chlorophyll-*a* fluorescence emission spectra (new Supplemental Fig. 4). We tentatively conclude that the primary cause of the production of singlet oxygen in OE_S plants is likely the strongly reduced amount of cyt b_6f (Table 1) in the strong overexpressors. The data are described on p.6-8 of our revised manuscript. Overall, our data suggest that, in the light, the strong overexpression causes pleiotropic effects, and the various physiological defects in these plants cannot be attributed to a single cause (see Discussion p.14). The major focus of our manuscript are, therefore, the consequences of PsbE operon overexpression in the dark, were clearly interpretable phenotypes are obtained.

11. The authors mention in the discussion that Cyt b-559 is a nucleation factor for PSII assembly and its presence in the prothylakoids could speed up the greening process. Yet, this seems to be the opposite of what is observed in the mutant. Some discussion needed.
We do not see accelerated greening in the overexpressors for two reasons. First, only a small proportion of the overexpressed PsbE forms active cytochrome b_{559}, and second, the secondary defects in the light preclude efficient biogenesis of the photosynthetic apparatus (see point 10 above).

Author actions necessary for acceptance
1. Confirmation that Cyt b-559 is formed and is present at elevated levels - Done (see point 2).
2. PTOX and NDH blots - Done for PTOX (Supplemental Figure 10A), technically not feasible for NDH (see point 5).
3. Determination of protein/lipid ratios - Done (Supplemental Figure 10B), see point 9.
4. Analysis of a second mutant in which a thylakoid protein has been over-expressed to test whether the phenotype is specific to psbEFLJ operon. - Experiments done (see point 8, and the explanation therein, why overexpression of single complex subunits does not result in protein overaccumulation).

Reviewer #2 (Comments for the Author):

Li and colleagues report engineering plastid "cytochrome b559 overexpressor tobacco plants", with the goal of understanding the role of the cytochrome in etiolated seedlings, where it accumulates in the WT context despite the absence of Photosystem II. b559 is a cytochrome of elusive function, while being part of the very core of Photosystem II across photosynthetic phyla. The premise the authors base their research on is interesting, and the question of the

PSII assembly and function timely. I have, however, strong reservations about the possibility of providing conclusions that the authors propose within their experimental framework. Given that the Photosystem II is degraded in the majority of mutants (including point mutations) of cyt b559, the authors design three overexpressor lines. They modified the chloroplast genome to increase the at both transcriptional and translational levels, acting on upstream elements of the entire psbE/F operon, and specifically on the 5' UTR of the first gene in the polycistronic product, psbE. The design and verification of these constructs and three overexpressor lines are solid, and include immunoblots against the gene products and other photosynthetic complexes, as well as northern blots to verify the transcript levels. The authors report tenfold increase in the PsbE accumulation than in the WT (although it looks more like a hundred-fold difference). Unfortunately, they do not report on the PsbF subunit of the apocytochrome, the first of three major experimental flaws of the paper, that in my opinion does not permit to draw the conclusions proposed by the authors. At that point, the article changes toward explanation of the bizarre phenotype observed in the PsbE overexpressing transplastomic plant, and despite essentially describing PsbE overexpressor continues to label the figures "strong cytochrome b559 overexpressor plants...". This is highly misleading, the holocytochrome b559 is comprised of PsbE, PsbF and the haem: neither of the latter two are present in increased amounts in the plants (or their presence was not verified). It is highly possible that even if PsbF was somehow upregulated in the OEs plants, the haem amount would be limiting for the accumulation of the holocytochrome, as no effort of boosting haem synthesis was made in these plants. The lack of PsbF co-overexpression, or haem synthesis enhancement - necessary steps in the process of "cytochrome b559 overexpression" - make it not acceptable to use such nomenclature in this manuscript, or draw conclusions about the cytochrome b559 role. Similarily, the discussion section states that the authors tried to address the function of cytochrome b559 by pursuing an overexpression approach - which is false, as in the best-case scenario they have attempted to overexpress two subunits of the apocytochrome.

*Furthermore, the overexpression misses at least one control: assuming that in etiolated OEs plants the PsbF level, as well as available haem levels follow the PsbE amount, reporting on any functional consequences of the cytochrome in, e.g., photoprotection requires a point mutant of one of the subunits of the cytochrome where the haem function is altered without a decrease of the PSII assembly and accumulation. This might be challenging given the previous unsuccessful trials of b559 mutagenesis. However, the authors also propose that PsbE (and not as they write "cyt. b559") controls, or exhibits important role in the deetionlation and/or PSII assembly. The evidence for that is the slightly lower rate of greening, and a membrane ultrastructure differences in the condition of very strong hydrophobic helix overaccumulation. This kind of claim clearly calls for an overexpression of other similar proteins to a similar extent, as the simple interpretation of the phenotype suggest that a mere presence of any excess hydrophobic molecule will result in an imbalance in proteostasis/membrane integrity. In other words, the link between the *specific* role of PsbE (let alone cyt. b559) is very much missing. It is indeed likely that the last sentence of the results section best encapsulates the findings (but needs a much stronger support that what is provided by the Authors): "...the abundance of membrane proteins in etioplasts limits prothylakoid biosynthesis, in that prothylakoids are continuously made until all synthesized proteins have been accommodated". This - rather than a specific role of PsbE (and even less so the holocytochrome b559) is what I suggest the authors try to prove.*

We fully agree with the Reviewer that overexpression of PsbE does not necessarily result in a corresponding overaccumulation of the cytochrome b_{559}. To be able to clarify the relationship between these two, we produced very large amounts of etiolated plant material to be able to isolate sufficient amounts of intact etioplasts from wild type and OE_S plants for spectroscopic quantification of the cytochrome. The quantification revealed a 1.44-fold increase of

cytochrome b_{559} in OE_S plants, in contrast to the approximately 10-fold increase in PsbE apoprotein accumulation (now accurately quantified – see new Fig. 4B). The data are included in Table 1 and discussed in the text on pp. 9-10 of the revised manuscript. We have now clarified throughout the manuscript that the effects we describe are predominantly due to overaccumulation of the PsbE protein in etioplast membranes. As suggested by the Reviewer, we have focused our manuscript and the conclusions drawn form our data on the relationship between membrane protein provision, lipid biosynthesis and prothylakoid proliferation. We did not intend to claim that this was a specific effect of cytochrome b_{559}, and apologize if this wasn't explained properly in the initial version of our manuscript.

Reviewer #3 (Comments for the Author):

*The authors of this paper pose the enigmatic question about the role of cyt b559 in PSII, which "has defied all attempts to elucidate its precise function in PSII function and biogenesis". Here, the authors focus on their structural role - in particular, during the biogenesis in angiosperms (here, tobacco) that do not synthesize chl in the dark but their etioplast / prolamellar body (PLB) contains cyt b559 but no PSII. Via generating psbE and psbF overexpressing (OE) tobacco, the authors provide evidence for a structural role of cyt b559 in PLB formation and prothylakoid biogenesis; their data also *strongly suggest* that OE of thylakoid membrane proteins regulates lipid biosynthesis in etioplasts. I think, these are important and valid conclusions - but with limitations, which should be clarified by the authors; also some corrections / amendments are requested.*

1/ Although the authors - correctly and carefully - draw their conclusions, I think for the 'general reader of TPC' it would be useful to emphasize that:

(i) These data do not give us the key to understand the structural and functional roles of cyt b559 in PSII, in general (e.g. in systems that do not synthesize PLB, or yet alone in cyanobacteria). I think considerations on these issues would be useful and some refinment of the text would help. For instance, the first sentence in Discussion may leave the false and evidently unintended impression that the authors obtained information about any particular structural/functional role of cyt b559. It can easily be envisioned that OE of other proteins present in etioplasts would lead to very similar effects. Also, as above, conclusions reached in the case of PLB and PTs won't explain the role of cyt b559 e.g. in cyanobacterial PSII. This should be clarified.

We agree and have revised the potentially misleading statements. In addition, we now clearly state that we cannot draw novel conclusions about the function of cyt b_{559} in the light (p.14: "The pleiotropic phenotypes (and the presence of a large fraction of free PsbE protein that is not assembled into functional cytochrome b_{559}) currently preclude novel conclusions about cytochrome b_{559} function in the light.").

We also agree with the Reviewer's assessment that the overexpression of other membrane proteins in etioplasts will likely lead to similar phenotypes, and have discussed this on p.15-16 of our revised ms.

(ii) Similarly, it remains to be answered or at least commented how much the effect of overexpression of cyt b559 might compare with OE of other membrane proteins. It is very likely but I do not see supporting data allowing us to conclude that "the abundance of thylakoid membrane proteins in etioplasts regulates lipid biosynthesis and, in this way, controls prothylakoid biogenesis". (I fully agree with the authors that "regulation of membrane

biogenesis by protein provision could be a more general theme in the biosynthesis of biological membrane systems.)

We discuss this in our revised manuscript as follows (p.15-16): "It would be interesting to overexpress other thylakoid membrane proteins in etioplasts and analyze the consequences at the ultrastructural level. However, our efforts to achieve overexpression of other thylakoid proteins have been unsuccessful. This is unsurprising, given that cytochrome b_{559} is the only small protein subcomplex known to accumulate in etioplasts. Although the NADH dehydrogenase complex, the ATP synthase and the cytochrome b_6f complex also accumulate in the dark, the individual subunits of these large complexes are not stable unless faithfully assembled into the complexes (e.g., Shikanai et al., 1998; Hager et al., 1999). Thus, overexpression of most, if not all, subunits of one of these complexes is likely required to obtain another case of prothylakoid membrane snailing."

Two examples of our efforts are shown in the figure above (see response to R#1, point 8). The data show the attempted overexpression of the b6f subunits PetA and PetN. As can be seen from panel A, no significant overexpression could be achieved (likely due to instability of the unassembled overexpressed subunits), and consequently, no thylakoid snailing is observed (panel B).

2/ Additional comments:

(i) Fv/Fm cannot and should not be equated with the maximum quantum efficiency of PSII - see Garab et al. 2023 JXB (and references therein). A more cautious and detailed analysis of ChlF is requested; the raw ChlF data would probably provide explanation on the nature of changes in PSII that lead to the observed decrease of Fv/Fm. E.g., to the present reviewer it appears very likely that OE of cyt b559 may lead to the accummulation of not fully matured PSII, with all - pleiotropic consequences (as in ll. 371-373). Thus, in the simplest case, instead of suggesting a decreased quantum efficiency, a mixture of active and inactive(d) PSII RCs might offer at least a phenomenologically correct explanation.

We agree and have rephrased this part accordingly. We have included the F0 values in Table 1, measured a number of additional parameters, including light response curves of several chlorophyll-a fluorescence parameters (new Supplemental Fig. 3), and also recorded 77K chlorophyll-a fluorescence emission spectra (new Supplemental Fig. 4) to better characterize the pleiotropic phenotypic consequences in the light. The data are described on p.6-8 of our revised manuscript.

(ii) l. 292: "PSII is the major site of 1O2 generation in the chloroplast" -a more detailed explanation is required. I presume the authors refer to the specific conditions of 1O2 generation via the formation of chl triplets. Please refine this section.

Done. Please see our response to comments to R#1, point 10.

(iii) In several cases (e.g. l. 311) the authors fail to add that significant increases are given relative to what.

Thanks for spotting this. These instances have been corrected.

Dear Ralph,

We have received the re-review of your manuscript entitled "Control of prothylakoid biogenesis by membrane protein provision in tobacco etioplasts." On the basis of the advice received, the board of reviewing editors would like to accept your manuscript for publication in The Plant Cell. This acceptance is contingent on revision based on the comments of our reviewer especially regarding the discussion points mentioned at the end of the reviewer's report (point 2 and 3). The reviewer also pointed out that the blot in Figure 4B is overloaded. However, if the signal of the loading series is linear, you do not need to re-do the blot. It would be sufficient to show the linearity in a graph and use the calibration curve to provide a quantitative relative estimate of the amount of the protein.

Please highlight all changes and include a detailed annotation of changes of the text, with line numbers, and noting your responses to the comments.

To submit your revised manuscript, click:

Link Not Available

If you have any questions about the revision submission procedures, please contact the Editorial Office Staff (tpc-submissions@aspb.org). If you cannot return the revised manuscript within 30 days, please let us know. Otherwise, we will assume that you have elected not to revise the manuscript and withdraw it.

Thank you very much for the privilege of reviewing this work. I look forward to receiving the next version.

Sincerely,

The Plant Cell Board of Editors

Reviewer #1 (Comments for the Author):

Importance of findings:
Cyt b-559 is known to play a physiologically important role in the assembly and maintenance of PSII in mature chloroplasts but the molecular mechanisms are still unclear and it remains quite possible that Cyt b-559 has functions outside PSII. The current submission links over-expression of PsbE to the proliferation of prothylakoids in etioplasts. This is a most interesting finding and will be of general interest to the readership of The Plant Cell.
Quality of experiments:
In general, a wide range of experiments have been performed and the data are of high quality.
Points in favour:
The novelty of the phenotype of the mutant etioplasts and wide range of experimental techniques used.
Points detracting:

1. The authors have now included new immunoblot data shown in Fig 4B to estimate the level of PsbE in OES. However, the data are not convincing as the blots are over-exposed, possibly because the authors have overloaded the gels. A dilution series of the OES sample shown in Fig 4A would suffice.
2. The authors have now confirmed that levels of Cyt b-559 are much lower than the level of PsbE apopolypeptide in etioplasts. It might be useful to mention the newly discovered Cyt b-559 assembly factor, RPH1, and that this might be limiting formation of Cyt b-559 (https://doi.org/10.1093/plcell/koae196)
3. The authors have confirmed enhanced transcript levels of the psbEFLJ operon in chloroplasts (Fig 3A) and etioplasts (Fig 4C) and enhanced levels of PsbE immunochemically (Fig 4A). But they have failed to determine whether levels of PsbL and PsbJ (and PsbF) are likewise elevated. Consequently, as mentioned previously, it seems premature to conclude that the effect on membrane ultrastructure is solely due to PsbE. The authors should make clear in the text that enhanced expression of PsbF, PsbL and PsbJ could contribute to the phenotype in etioplasts. This is especially important in the abstract which potentially gives the misleading impression that PsbE is solely responsible.
4. The authors show in Fig 5C that there is substantial membrane proliferation in the OES mutant (approx. 3-fold more on a length basis). Yet it seems from quantification of lipid that there is only 25% more lipid in the OES mutant (Fig 7) for each class. One would expect considerably more lipid in the OES mutant given the similar protein/lipid ratio and the greater amount of membrane. These observations seem somewhat counter intuitive and need clarification.
5. Were the ultrastructure changes observed in the OES mutant maternally inherited?
6. Page 10: immunoblots do not give hybridization signals

Author actions necessary for acceptance
1. Redo blot shown in Fig 4B so that signals can be clearly quantified.
2. Make clear that PsbF, PsbL and PsbJ might be over-expressed.
3. Insert discussion points

Please also note the following:

-The Plant Cell now requires authors to complete and submit an author revisions checklist upon submission of a revised manuscript. The aim of the checklist is to aid authors in preparing a high-quality manuscript, facilitate the review and assessment of revised manuscripts, and help to ensure that journal standards are maintained across the board. If your manuscript is accepted, the completed checklist will be published as supplemental material attached to the article online. Please download a copy of the checklist (pdf fillable form) at this link, for submission with your revised manuscript: https://tpc.msubmit.net/html/Author_Revisions_Checklist.pdf.

-Supplemental materials should be restricted to large datasets and tables, presentation of replicates, and validation of reagents, methods, or genotypes. Any data that are used to support the major claims must be in the main manuscript. Supplemental figure legends must indicate what figure in the main manuscript is supported by the supplemental data presented. Please justify how each of the supplemental figures meet the criteria.

-Sampling methods and the nature of "biological replicates" should be described precisely (i.e. different plants, parts of plants, pooled tissue, independent pools of tissue, sampled at different times, etc.), along with a clear description of and rationale for any statistical analyses conducted. The reader should know exactly what was sampled; what forms the basis of the calculation of any means and statistical parameters reported. This is also necessary to ensure that proper statistical analysis was conducted.

Point-by-point response to the Editor's and Reviewer's suggestions (with our response in blue)

Editor:
This acceptance is contingent on revision based on the comments of our reviewer especially regarding the discussion points mentioned at the end of the reviewer's report (point 2 and 3). The reviewer also pointed out that the blot in Figure 4B is overloaded. However, if the signal of the loading series is linear, you do not need to re-do the blot. It would be sufficient to show the linearity in a graph and use the calibration curve to provide a quantitative relative estimate of the amount of the protein.
Done – see response to reviewer below. As suggested, we have quantified the immunoblot data and the results clearly show that the three values used for PsbE protein quantitation are well within the linear range of the signals of the dilution series (revised Fig. 4B).

Reviewer #1 (Comments for the Author):

1. The authors have now included new immunoblot data shown in Fig 4B to estimate the level of PsbE in OES. However, the data are not convincing as the blots are over-exposed, possibly because the authors have overloaded the gels. A dilution series of the OES sample shown in Fig 4A would suffice.
As suggested by the Editor, we now have quantified the immunoblot data and the results clearly show that the three values used for PsbE protein quantitation are well within the linear range of the signals of the dilution series (revised Fig. 4B). As can be seen from the graphs, both the three signals from wild-type loading series (10x,15x and 20x) and the first three signals (1x, 2x and 3x) from the OE_S plants are in the linear range (whereas the 4x OE_S is not due to overloading, but this value has not been used for quantification). Text changes are briefly described in the legend to the revised figure.

2. The authors have now confirmed that levels of Cyt b-559 are much lower than the level of PsbE apopolypeptide in etioplasts. It might be useful to mention the newly discovered Cyt b-559 assembly factor, RPH1, and that this might be limiting formation of Cyt b-559 (doi.org/10.1093/plcell/koae196).
We thank the Reviewer for drawing our attention to this new paper. RPH1 seems to be responsible for the accumulation of cytochrome b_{559} during the early steps of PSII biogenesis, possibly by facilitating heme insertion into the apoprotein. We now briefly discuss its possible limiting role in the Discussion section (lines 464-468).

3. The authors have confirmed enhanced transcript levels of the psbEFLJ operon in chloroplasts (Fig 3A) and etioplasts (Fig 4C) and enhanced levels of PsbE immunochemically (Fig 4A). But they have failed to determine whether levels of PsbL and PsbJ (and PsbF) are likewise elevated. Consequently, as mentioned previously, it seems premature to conclude that the effect on membrane ultrastructure is solely due to PsbE. The authors should make clear in the text that enhanced expression of PsbF, PsbL and PsbJ could contribute to the phenotype in etioplasts. This is especially important in the abstract which potentially gives the misleading impression that PsbE is solely responsible.
We agree and now explicitly mention this possibility (lines 476-479).

4. The authors show in Fig 5C that there is substantial membrane proliferation in the OES mutant (approx. 3-fold more on a length basis). Yet it seems from quantification of lipid that there is only 25% more lipid in the OES mutant (Fig 7) for each class. One would expect considerably more lipid in the OES mutant given the similar protein/lipid ratio and the greater amount of membrane. These observations seem somewhat counter intuitive and need clarification.
We have shown that total protein : total lipid ratios remain unaltered. We do not think that one should expect a larger increase in lipids than the observed 25% increase, given that the etioplast lipids make up only a moderate part of the total cellular lipids.

5. Were the ultrastructure changes observed in the OES mutant maternally inherited?
Yes, we have shown that, as expected, the OE_S mutant phenotype is maternally inherited (Fig. 1C and Supplemental Fig. S1).

6. Page 10: immunoblots do not give hybridization signals
Agreed, and changed to “PsbE signals”.

Dear Ralph,

We are pleased to inform you that your paper entitled "Control of prothylakoid biogenesis by membrane protein provision in tobacco etioplasts" has been accepted for publication in The Plant Cell, pending a final editorial review by a science editor. At this stage, your manuscript will be evaluated by a science editor with respect to its presentation of scientific content, compliance with journal policies, and presentation for a broad readership. The Plant Cell has contracted with Plant Editors (planteditors.com) to provide this service to our authors, and you will soon receive additional information on this process.

Please note that each author needs to link their ORCID identifier to their account in the system before your manuscript can be published. If any authors do not have an ORCID linked to their account, they will receive a message with a link to complete this task. Please ensure that ALL of your coauthors have completed this task as soon as possible.

LICENSE INFORMATION Shortly after your paper is sent to production, you will receive an email from our publisher's author support team, **SciPris**, with information on article processing charges, as well as the types of Open Access license you would be able to purchase. The accepted version of your manuscript will not appear on our Advance Articles page until the license is selected and signed, so please look out for an email from SciPris. For more information see the Article Publication Fees section of our author guidelines: https://academic.oup.com/plcell/pages/General-Instructions

The Plant Cell and The Arabidopsis Information Resource (TAIR) are collaborating to collect functional annotation data about Arabidopsis genes from authors. This includes information about the gene's molecular function (e.g., kinase activity, ATP synthetase activity), the biological process/es it is involved in (e.g., endosperm development, threonine biosynthesis), its subcellular location (e.g., nucleus, ER), anatomical or developmental expression pattern (e.g., leaf, ovule, flower stage 10, seedling stage), or its partner in a protein-protein interaction (e.g., AT1G01010 interacts with AT1G01020).

If your paper contains results falling into one or more of these categories for Arabidopsis genes, we request that you now submit these data for inclusion in TAIR by filling in the form provided at the following URL: https://www.arabidopsis.org/doc/submit/functional_annotation/123. If you need further clarification on what types of data can be submitted please contact curator@arabidopsis.org.

Finally, we encourage your submission of artwork for the journal cover. If there is an image or illustration related to your paper that you would like to have considered, please email it in .pdf or .tif format to clowe@aspb.org. An image title, brief, 2-3 sentence description of the image, and the name of the person credited for the image are also encouraged. If your image is selected to appear on the online cover of the journal, you will be contacted with the scheduled issue date and further information.

We look forward to seeing your paper published.

Sincerely,

The Plant Cell Board of Editors

=====
IMPORTANT REMINDER: PEER REVIEW REPORTS
=====

If you opted to publish a peer review report along with your article during the original submission process, it will be prepared by the editorial staff and publicly posted with your manuscript, inside the zip file that contains any other supplemental material. As a reminder, the peer review report is a public record of all comments from editors and reviewers, as well as your prior responses, as you received them in the decision letters for each draft of your manuscript. If you agreed to publish this report and have changed your mind, or are not sure if you selected this option, please contact the editorial office as soon as possible before signing the license agreement from our publisher.

----FOR ASPB OFFICE USE ONLY (DO NOT EDIT)----
MSID: 36786
Corresponding Author: Prof. Dr. Bock
